# Multi-feature Response Attention Network for kidney stone detection and measurement of stone size in ultrasound images

**DOI:** 10.1371/journal.pone.0359214

**Published:** 2026-10-15

**Authors:** Vijaya Padmanabha, Mohamed Sirajudeen Yoosuf, Anand Mahendran, Joseph Mani, Anisha Prima Rodrigues, Gururaj Bijur, Hothefa Jassim

**Affiliations:** 1 Department of Mathematics and Computer Science, Modern College of Business and Science, Bowshar, Muscat, Sultanate of Oman; 2 Intelligent Computing and Business Systems (ICBS), St. Joseph Engineering College, Mangalore, India; 3 Manipal Institute of Technology, Manipal Academy of Higher Education, Manipal, India; Chongqing Medical University, CHINA

## Abstract

Kidney stones are hard deposits formed in the urinary tract due to excess waste and low fluid intake, causing severe abdominal pain. Ultrasound-based kidney stone detection involves identifying and locating stones in the kidneys using high-frequency sound waves to visualize renal structures. Measuring stone size is crucial for early treatment and preventing infection. However, detection is challenging because ultrasound images suffer from noise, lower contrast, and variable quality. To overcome these limitations, the Multi-feature Response Attention Network (MFRANet) is proposed to detect kidney stones. Initially, the input image is allowed for image denoising and edge preservation using Windowed Variation Kernel Wiener Filter (WV-KWF). Further, the kidney region is segmented by the Fuzzy Clustering algorithm with Variable Multi-pixel Fitting spatial information (FCM-VMF). Moreover, descriptor computation is processed by rotated Binary Robust Independent Elementary Feature (rBRIEF) with Discrete Cosine Transform (DCT). Afterwards, the kidney stone is detected using MFRANet, wherein MFRANet is designed by combining Multi-feature Multi-Attention Network (MFMANet), along with Group Response Attention Network (GRA-Net). Next, the detected stone is segmented using Optimized DA-TransUNet (O-DAT), and lastly, the kidney stone size is measured. Performance of the proposed MFRANet is assessed with Accuracy, F1-score, Precision, and True Positive Rate (TPR) which achieved superior values of 96.64%, 96.65%, 97.44%, 95.88% Response Attention Network, Multi-feature Multi-Attention Network, Optimized DA-TransUNet.

## 1. Introduction

Kidney disorders involve a gradual decline in renal health, often accompanied by alterations in kidney structure and function arising from diverse pathological factors. Among its most significant forms is chronic kidney disease (CKD), clinically defined by a sustained reduction in kidney function, typically indicated by an estimated Glomerular Filtration Rate (eGFR) or by the presence of markers, such as albuminuria, haematuria, or imaging abnormalities persisting for at least three months [1]. CKD represents a major global health concern which disturbs nearly 10 percent of population worldwide, accounting for an estimated 1.2 million deaths per annum. By 2040, CKD will become the fifth leading cause of death globally, reflecting one of the most significant increases among major causes of mortality [2]. Kidney stone disease, termed as urolithiasis, is a prevalent as well as increasingly common urological syndrome with incidence rates rising globally. Its occurrence varies regionally, influenced by factors, such as diet, lifestyle, and environmental conditions. Despite advances in both medical and surgical management, kidney stones continue to pose considerable clinical challenges [3]. Ultrasonography has emerged as a crucial non-invasive diagnostic modality for detecting kidney stones. It provides real-time visualization without radiation exposure, making it especially suitable for pregnant women and children. Ultrasound imaging facilitates the assessment of stone presence, size, and location, as well as complications, like hydronephrosis. Furthermore, therapeutic strategies including extracorporeal shock wave lithotripsy, ureteroscopy, and percutaneous nephrolithotomy are often guided by imaging findings, which play a central role in optimizing treatment selection and improving patient outcomes [4].

Besides kidney stones, other structural irregularities, like renal cysts as well as tumours, affect kidney function and health significantly. Renal cysts, which are fluid-filled sacs, develop within kidney tissue, enlarge over time, compress surrounding nephrons, and ultimately impair renal performance [5]. Polycystic Kidney Disease (PKD) is inherited renal disorder in which numerous cysts develop within kidneys, gradually impairing their normal function and potentially resulting in advanced renal failure. In contrast, Renal Cell Carcinoma (RCC) is a major malignant tumor originating in kidney and represents the predominant type of kidney cancer, which is a malignant tumour responsible for a considerable proportion of cancer-related deaths worldwide. Early detection and effective management of such irregularities are crucial in preserving the function of the kidney, avoiding complications, and improving patient outcomes. Computer-aided diagnostic systems have emerged as a promising solution for early detection and diagnosis for increasing global burden of kidney diseases and the shortage of specialized healthcare professionals, particularly nephrologists [6]. Machine Learning (ML) algorithms and Artificial Intelligence (AI) help clinicians to identify kidney abnormalities with greater accuracy and efficiency. Kidney stone disease remains particularly common and is often associated with severe pain and potentially serious complications. As stones enlarge, they cause haematuria, dysuria, nausea, and sharp pain in lower abdomen, especially as they migrate through the urinary tract. The delayed detection led to a decline in kidney function, reduced life’s quality, life-threatening complications. Therefore, the early and accurate diagnosis of kidney stones and other renal abnormalities is essential for effective treatment planning and long-term disease management [7].

Among various imaging modalities, ultrasonography remains a widely used, non-invasive, cost-effective, and radiation-free technique for the initial assessment of kidney stones [8]. However, the ultrasound imaging is highly operator-dependent and often affected by noise, variability, and limited visibility of small or obstructed stones, which hinder consistent and accurate diagnosis. Hence, DL-based approaches, namely Convolutional Neural Networks (CNNs), are increasingly used for kidney stone identification in ultrasound images. These models learn intricate spatial and textural patterns directly from raw image data, lowering the need for feature extraction manually or traditional image pre-processing [9]. Recent AI-based studies have demonstrated the potential of Deep Learning (DL) for the ultrasound-based detection of kidney stone, classification, prediction, and clinical management. However, recent reviews on deep-learning-based medical image segmentation have highlighted that limited annotated datasets, imaging-protocol variability, lesion heterogeneity, and insufficient external validation remain major challenges affecting clinical applicability. These challenges emphasize the need for robust and generalizable AI frameworks with reliable clinical evaluation for kidney stone analysis. Therefore, developing robust and generalizable AI frameworks is essential for reliable kidney stone diagnosis and management. Although the Non-Contrast Computed Tomography (NCCT) remains the diagnostic gold standard because of its superior sensitivity, DL-enhanced ultrasound presents a valuable alternative for real-time, radiation-free screening, particularly in resource-limited or radiation-sensitive populations. As the global prevalence of kidney stone disease continues to rise, the adoption of AI-driven ultrasound systems offers substantial promise for facilitating early, accurate, and accessible diagnosis, ultimately improving clinical outcomes and alleviating the burden on healthcare systems [10,11].

This work introduces the proposed MFRANet for detecting stones in the kidney. At initial stage, input images from dataset undergo noise reduction and edge-preserving enhancement using WV-KWF method. Afterwards, the segmentation process in the kidney region is done by FCM-VMF. Next, descriptor computation is employed by rBRIEF with DCT. Moreover, kidney stone detection is carried out by MFRANet, wherein MFRANet is developed by merging MFMANet and GRA-Net. Next, the detected stone label and segmented output are used for kidney stone segmentation, which is done using O-DAT, and at last, the size of the kidney stone is measured.

The newly developed contribution used in this paper is stated below,

- **Proposed MFRANet for detecting kidney stones:** Kidney stone detection is difficult because of its smaller size and variable shape. Here, a kidney stone is detected by MFRANet, where MFMANet and GRA-Net are combined to form this newly developed network.

The paper is organized as follows: Section 2 reviews existing and conventional approaches, Section 3 presents proposed MFRANet framework for kidney stone detection, Section 4 discusses the experimental results and their analysis, and Section 5 concludes the study.

## 2. Motivation

Kidney stones are a condition leading to obstruction, infection, or kidney damage if not detected in time. While CT scans provide high accuracy, they expose patients to ionizing radiation and are expensive and less available in resource-limited settings. Ultrasound imaging is non-invasive, radiation-free, portable, and widely available, making it ideal for routine screening and diagnosis. However, manual interpretation of ultrasound images remains challenging because of noise, low contrast, and operator dependency. Therefore, developing reliable approaches for detecting kidney stones in ultrasound images is highly motivated to improve diagnostic accuracy, eradicate human error, and support early, efficient, and radiation-free diagnosis of kidney stones.

### 2.1. Literature survey

Y. A. Asaye, *et al.* [12] developed KNN for detecting kidney stones from ultrasound images. This model automatically analyzed ultrasound images, reducing the dependence on human interpretation. However, this model lacked external validity since did not tested on more different database, representing both local and global populations. G. Macin, *et al.* [13] designed KidneyNeXt for renal tumor classification. KidneyNeXt demonstrated better potential and formed a decision support tool in the diagnosis of tumors in the kidney. However, extra validation on huge datasets and integrating clinical settings did not contribute meaningfully towards early detection and treatment planning. P. Kumar, *et al.* [14] modelled a CNN/ResNet model for kidney stone detection. This model automatically extracted intricate features from medical images, allowing it to detect subtle patterns and abnormalities. But, the CNN/ResNet model performed poorly when images had noise, artifacts, or variations in imaging protocols, machine types, or resolutions. A. N. Jadhav, *et al.* [15] designed a hybrid CNN-Long Short-Term Memory (LSTM) model for enhancing detection of kidney stones. This hybrid model detected subtle abnormalities in early-stage images that may be missed by traditional methods or radiologists. But this approach required continuous retraining and validation for new imaging devices or patient populations.

S. Asif, *et al.* [16] presented PSOWeightedAvgNet for kidney stone detection. This approach provided high interpretability and delivered valuable insights for informed treatment planning. However, its generalizability was limited due to a lack of evaluation on diverse datasets. J. Chaki and A. Ucar [17], modelled an inductive transfer-based ensemble Deep Neural Network (DNN) for detecting kidney stones. It demonstrated reliability and strong decision-making capabilities. Nevertheless, this model did not assess its effectiveness in diagnosing different categories of tumors, limiting its potential clinical utility as well as generalizability. S. L. Rapelang and I. C. Obagbuwa [18], modelled a hybrid CNN-SVM for detection of kidney stones. This model achieved enhanced diagnostic accuracy and successfully captured complex, high-level features. Nevertheless, it did not investigate advanced optimization methods for effective hyperparameter tuning. C. Yan and N. Razmjooy [19], presented a Deep Believe Network (DBN) for kidney stone detection. Even though DBN automatically learnt deep hierarchical representations of medical images without extensive manual feature engineering, it remained computationally intensive and required a long training time.

### 2.2. Challenges

Issues faced by current techniques associated with kidney stone detection are given below:

- ML models, such as KNN [12], detected subtle texture patterns or intensity variations that were not easily visible to the human eye for kidney stone detection. However, the accuracy of KNN heavily depends on the quality of ultrasound images, which vary due to operator skill, patient body type, or equipment settings.
- CNN/ResNet in [14] significantly enhanced diagnostic accuracy for kidney stone detection, demonstrating superior performance compared to traditional methods. However, this approach lacked transparency in its decision-making process, which limited its acceptance and integration within clinical workflows.
- PSOWeightedAvgNet in [16] for kidney stone detection achieved promising results, improving reliability across diverse clinical datasets. Nevertheless, the absence of comprehensive error case analysis restricted the deeper understanding of misclassifications, thereby limiting opportunities for model refinement and error reduction.
- In [17], an inductive transfer-based ensemble DNN created a richer and more discriminative feature set, improving classification and segmentation performance. Despite its effectiveness, this model did not sufficiently address the challenge of overfitting, that affect generalization capability of the model.
- CKD represents a major global health challenge, often leading to progressive loss of renal function and potential failure in kidney. Kidney stones are one of the contributing issues, which further impair kidney performance.

## 3. Proposed multi-feature response attention network for kidney stone detection

Accurate detection of kidney disease is essential, as this represents a major healthiness concern that requires timely and precise detection to ensure efficient treatment and prevent serious complications. Moreover, identifying the size of the stone is essential for an accurate treatment process. However, existing models often face challenges, such as overfitting and limited accuracy, particularly when trained on imbalanced datasets. To tackle these complications, this research proposes MFRANet for kidney stones detection in ultrasound images. Initially, the ultrasound kidney image taken from a specific dataset illustrated in [20–22] is allowed for image denoising and edge preservation utilizing WV-KWF [23] to reduce speckle noise while preserving important kidney and stone boundaries. Then, kidney region segmentation is done based on FCM-VMF [24] to extract the kidney region and minimize interference from surrounding tissues. Further, descriptor computation is processed by rBRIEF [25] with DCT [26] to obtain robust feature representations by capturing local texture characteristics and frequency-domain information. Next, kidney stone detection is done using MFRANet, where MFRANet is developed by combining MFMANet [27] and GRA-Net [28]. Here, the detected output is classified as stone and non-stone. Next, the O-DAT [29] is employed to accurately segment the detected kidney stone region and facilitate precise stone size measurement. The proposed framework is mainly designed for ultrasound kidney stone analysis, where WV-KWF and rBRIEF+DCT address noise and feature extraction challenges. CT datasets are used for complementary evaluation and segmentation analysis due to their clear structural information.

Fig 1 shows a block diagram of MFRANet for kidney stone detection in ultrasound images.

**Fig 1:**
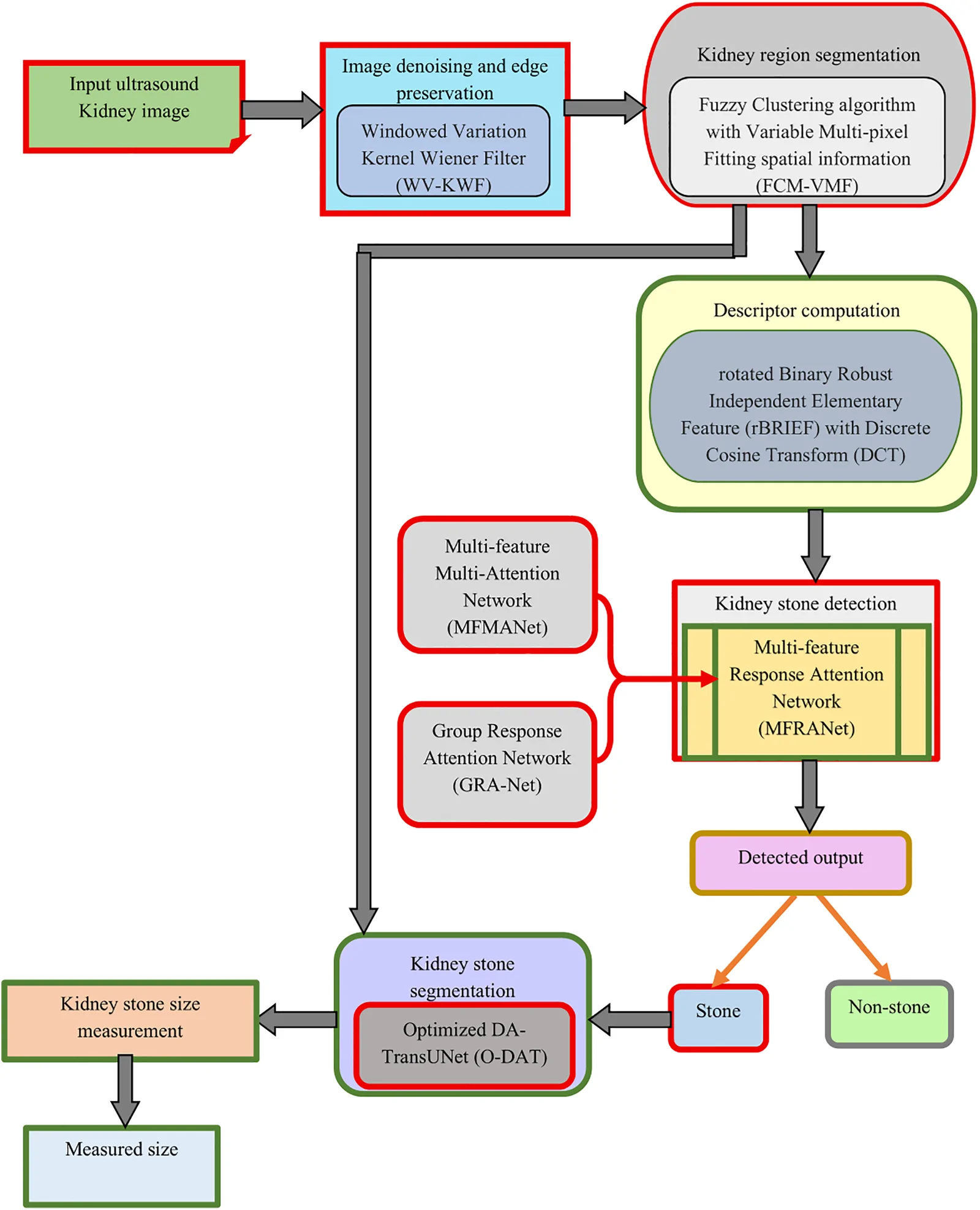
Block diagram of MFRANet for kidney stone detection in ultrasonic images.

### 3.1. Acquisition of ultrasound kidney image from kidney image datasets

The datasets used are the kidney-stone-ultrasound Computer Vision Model dataset [20], KSSD2025- Kidney Stone Segmentation Dataset [21], and CT Kidney Dataset: Normal-Cyst-Tumor and Stone [22]. The kidney-stone-ultrasound Computer Vision Model dataset [20] comprises 5431 total images with a train set, valid set, and test set. This dataset is indicated below,


$$\begin{array}{c} Q=\left\{Q_{\text{1}},Q_{\text{2}},...,Q_{\beta },...,Q_{m}\right\} \end{array}$$ (1)

KSSD2025 [21] is a collection of axial CT scans accompanied by expert-annotated kidney stone segmentation masks, designed to advance DL research in medical image segmentation, from which the kidney stone images are taken for analysis. Simultaneously, the CT Kidney Dataset [22] was gathered from Picture Archiving and Communication Systems (PACS) across multiple hospitals in Dhaka, Bangladesh, comprising cases of patients previously diagnosed with kidney tumors, cysts, normal conditions, or stones. From the CT Kidney Dataset, the normal kidney images are taken for analysis. Finally, the abnormal images from KSSD2025 and normal images from the CT Kidney Dataset are combined to form newer dataset, which is expressed as below,


$$\begin{array}{c} R=\left\{R_{\text{1}},R_{\text{2}},...,R_{j},...,R_{n}\right\} \end{array}$$ (2)

Herein, Q and R indicates kidney-stone-ultrasound Computer Vision Model dataset and the newly formed dataset from KSSD2025, along with the CT Kidney Dataset. $Q_{m}$ and $R_{n}$ specify the total images present in both datasets, $Q_{\beta }$ and $R_{j}$ are the input images taken from the datasets. The image $Q_{\beta }$ from kidney-stone-ultrasound Computer Vision Model dataset is used as input for the process below, where the same procedure is followed for $R_{j}$ also.

### 3.2. Image denoising and edge preservation using WV-KWF

Edge-preserving denoising of ultrasound images reduces unwanted speckle interference while maintaining important anatomical contours and subtle structural details. The WV-KWF [23] is applied in this process by adapting Wiener filtering locally. The ultrasound input image $Q_{\beta }$ is allowed for the process of denoising and edge preservation. First, the kernel or weighting function is computed on patch β. This kernel reflects locality, or similarity information, to weigh contributions from nearby patches. Then, quantities like the Fourier transform of $\alpha_{\beta }$, such as $A_{\beta ,\chi }$, and the Fourier transform of $\delta_{\beta }\circ {\overset{\mathrm{\backslash lower}0.5em\mathrm{\backslash smash}⌢}{\}}Q}_{\beta }$, such as $B_{\beta ,\chi }$, are computed. Based on these parameters, the denoised pixel value $x_{\beta }$ is computed, which is derived as below,


$$\begin{array}{c} x_{\beta }=\frac{\text{1}}{a^{\text{2}}}\sum_{\chi \in \Delta_{\beta }}A_{\beta ,\chi }B_{\beta ,\chi } \end{array}$$ (3)

Here, $\Delta_{\beta }$ specifies the local frequency domain, χ indicates the coordinate in the frequency domain, and the noisy pixel image $Q_{\beta }$ is of size a×a. Then, the weighted average of $x_{\beta }$ is aggregated for each pixel in the image to form the single denoised image $X_{\beta }$, at which the edges of the image are also preserved. The implementation parameters of WV-KWF were selected considering the characteristics of ultrasound images. A 7 × 7 adaptive window was used for kernel computation, assuming multiplicative speckle noise in ultrasound images. Filtering parameters were optimized through grid-search strategy combined with five-fold cross-validation. Denoising parameters were optimized using complete dataset rather than only the training data; therefore, the same optimized parameters were applied during all experimental evaluations.

### 3.3. Segmenting kidney region using FCM-VMF

It is separated from surrounding tissues, such as liver, spleen, fat, etc. Here, FCM-VMF [24] is used for segmenting the kidney region, where FCM-VMF is a novel fuzzy clustering algorithm designed to improve image segmentation by incorporating spatial information adaptively. The denoised image $X_{\beta }$ is used as input for segmenting regions in the kidney. FCM-VMF is designed by presenting an updated neighborhood space constraint parameter $\sum_{b=1}^{d}\sum_{c=1}^{e}{\mathbb{R}}_{bc}^{f}{\|{ℵ}_{c}-{\mathbb{Z}}_{b}\|}^{\text{2}}$ into FCM’s objective function, which is given as below,


$$\begin{array}{c} \mathbb{C}=\sum_{b=1}^{d}\sum_{c=1}^{e}{\mathbb{R}}_{bc}^{f}{\|{\mathbb{Q}}_{c}-{\mathbb{Z}}_{b}\|}^{\text{2}}+\varphi \sum_{b=1}^{d}\sum_{c=1}^{e}{\mathbb{R}}_{bc}^{f}{\|{ℵ}_{c}-{\mathbb{Z}}_{b}\|}^{\text{2}} \end{array}$$ (4)

Herein, f specifies the weighting exponent, d resembles cluster counts, e indicates pixel counts, ${\mathbb{Z}}_{b}$ indicates $b^{th}$ cluster center, ${ℵ}_{c}$ indicates filtered value of $c^{th}$ pixel in improvised filtered image, ${\mathbb{R}}_{bc}$ resembles membership degree of pixel ${\mathbb{Q}}_{c}$ to $b^{th}$ cluster center, and φ resembles parameter controlling multi-pixel fitting spatial neighborhood parameter effect. FCM-VMF’s algorithmic steps are as follows,

***Step 1:*** Consider the count of clusters as d(2<d<e), maximal iteration counts as $P_{max}$, threshold as γ, weighted exponent as f, multi-pixel fitting spatial information term φ, variable multi-pixel fitting term as $\eta_{in}$, generalized neighborhood window as h×h, and dimension of filter window as g×g.

The parameters of FCM-VMF were selected empirically. Number of clusters was 3, membership threshold was 0.5, and a 5×5 spatial neighborhood window was used for incorporating local spatial information. The iterative optimization was terminated when it is lower than $1\times 10^{-5}$ or when the maximum iteration limit of 100 was reached.

***Step 2:*** Classify input image $X_{\beta }$ to multiple filter windows and positions of every filter window based on the window variable approach.

***Step 3:*** Gain enhanced filtered image ℵ based on the equations,


$$\begin{array}{c} {\mathbb{Q}}_{b,c,P}=g_{\text{1}}b+g_{\text{2}}c+g_{\text{3}} \end{array}$$ (5)

The above equation is achieved by fitting pixels in their equivalent generalized neighborhood window, generalized neighborhood window is considered as 2-dimensional plane. Reference value in relation to pixel ${\mathbb{Q}}_{b,c}$ is ${\mathbb{Q}}_{b,c,P}$, abscissa of generalized neighborhood window is b. The corresponding filtered image ${ℵ}_{b,c}$ is estimated as below,


$$\begin{array}{c} {ℵ}_{b,c}=\frac{{\mathbb{Q}}_{b,c}+\Psi_{b,c}{\mathbb{Q}}_{b,c,P}}{1+\Psi_{b,c}} \end{array}$$ (6)

Herein, the weighting factor is $\Psi_{b,c}$. This is formed by considering control factors $\Phi_{b,c}$ and $\iota_{b,c}$.

***Step 4:*** Cluster centers are initialized and the count of iterations P=1.

***Step 5:*** Membership ${\mathbb{R}}_{bc}^{\left(P+1\right)}$ function is updated using the equation,


$$\begin{array}{c} {\mathbb{R}}_{bc}=\frac{\text{1}}{\sum_{\kappa =1}^{d}{\left(\frac{{\|{\mathbb{Q}}_{c}-{\mathbb{Z}}_{b}\|}^{\text{2}}+\varphi {\|{ℵ}_{c}-{\mathbb{Z}}_{b}\|}^{\text{2}}}{{\|{\mathbb{Q}}_{c}-{\mathbb{Z}}_{\kappa }\|}^{\text{2}}+\varphi {\|{ℵ}_{c}-{\mathbb{Z}}_{\kappa }\|}^{\text{2}}}\right)}^{\frac{\text{1}}{\left(f-1\right)}}} \end{array}$$ (7)

***Step 6:*** The cluster center ${\mathbb{Z}}_{b}^{\left(P+1\right)}$ is updated using the formula,


$$\begin{array}{c} {\mathbb{Z}}_{b}=\frac{\sum_{c=1}^{e}{\mathbb{R}}_{bc}^{f}\left({\mathbb{Q}}_{c}+\varphi {ℵ}_{c}\right)}{\sum_{c=1}^{e}\left(1+\varphi \right){\mathbb{R}}_{bc}^{f}} \end{array}$$ (8)

***Step 7:*** If $\|{\mathbb{N}}^{\left(P+1\right)}-{\mathbb{N}}^{\left(P\right)}\|<\gamma$ or $P>P_{max}$, then stop; else P=P+1, return to step 5. Thus, the kidney region is segmented and the attained output from this process is indicated as $S_{\beta }$.

### 3.4. Descriptor computation by rBRIEF with DCT

Descriptor computation refers to the process of extracting numerical features that uniquely represent important characteristics of an image region. Although MFRANet can automatically learn features, rBRIEF and DCT are incorporated to enhance feature representation in ultrasound images by capturing rotation-invariant texture patterns and frequency-domain information, improving the detection of small and low-contrast kidney stones. Here, descriptor computation is employed using rBRIEF [25] with DCT [26]. The kidney region-segmented image $S_{\beta }$ is allowed as input to rBRIEF to get a textural image $Tex_{\beta }$. These band images are concatenated to get the feature vector $K_{\beta }$.

#### 3.4.1. rBRIEF descriptor.

The input given to the rBRIEF descriptor [25] is a kidney region segmented image $S_{\beta }$. For matching the feature points attained in successive frames, the rBRIEF descriptor is utilized for measuring similarity. In case of smoothed image patch $P_{ip}$, binary test criteria l is formulated as below for constructing a binary string.


$$\begin{array}{c} l\left(P_{ip};n_{\text{1}},n_{\text{2}}\right)=\left\{ \begin{array}{c} @l@1\text{\textbackslash{}hspace\{0.17em}\;\}if\text{\textbackslash{}hspace\{0.17em}\;\}G_{n_{\text{1}}}<G_{n_{\text{2}}}\\ 0\text{\textbackslash{}hspace\{0.17em}\;\}otherwise \end{array}\right. \end{array}$$ (9)

Wherein, the grayscale of point $n_{\text{1}}=\left(x_{\text{1}},y_{\text{1}}\right)$ and $n_{\text{2}}=\left(x_{\text{2}},y_{\text{2}}\right)$ on patch $P_{ip}$ are $G_{n_{\text{1}}}$ and $G_{n_{\text{2}}}$. After selection of t pairs of pixels from patch $P_{ip}$ for carrying out binary test.


$$\begin{array}{c} \lambda_{t}\left(P_{ip}\right)=\sum_{\begin{array}{c} 1\leq w\leq t\\ 1\leq o\leq t\\ n_{w},n_{o}\in P_{ip} \end{array}}l\left(P_{ip};n_{w},n_{o}\right) \end{array}$$ (10)

Feature from Accelerated Segment Test (oFAST) feature point is taken as important as the BRIEF feature. The 2×t undirected description matrix is developed using $\left(x_{w},y_{w}\right)$ of any feature set.


$$\begin{array}{c} D=\left(\begin{array}{ccccc} @ccccc@x_{\text{1}} & x_{\text{2}} & x_{\text{3}} & \cdots & x_{w}\\ y_{\text{1}} & y_{\text{2}} & y_{\text{3}} & \cdots & y_{w} \end{array}\right) \end{array}$$ (11)

Affine matrix $A_{\varpi }$ is indicated based on the direction ϖ of oFAST feature point.


$$\begin{array}{c} A_{\varpi }=\left[\begin{array}{cc} @cc@cos\varpi & sin\varpi \\ -sin\varpi & cos\varpi \end{array}\right] \end{array}$$ (12)

A novel directional description matrix is attained by revolving matrix D with ϖ direction.


$$\begin{array}{c} D_{\varpi }=A_{\varpi }D \end{array}$$ (13)

Based on Eq. (10) and Eq. (13), the rBRIEF descriptor with rotational invariance is designed by


$$\begin{array}{c} i_{\varpi }\left(P_{ip},\varpi \right)=\lambda_{t}\left(P_{ip}\right)|\left(x_{w},y_{w}\right)\in D_{\varpi } \end{array}$$ (14)

Here, the textural image $Tex_{\beta }$ is attained after application of the rBRIEF descriptor.

#### 3.4.2. DCT.

The attained textural image $Tex_{\beta }$ is applied to DCT [26], where DCT is a mathematical transform converting pixel values in the image from the spatial domain to the frequency domain. The DCT expresses the image as a sum of cosine waves of multiple frequencies, indicating low frequency as smooth areas, and high frequencies as edges or finer details. The formula indicating 2D-IDCT, formed by extending 1D-DCT for images, is expressed as below,


$$\begin{array}{c} H\left(u,v\right)=\frac{\text{2}}{\sqrt{U\times V}}\sum_{b=0}^{U-1}\sum_{j=0}^{V-1}E\left(b\right)E\left(j\right)F\left(b,j\right)cos\frac{(2u+1)b\pi }{2U}cos\frac{(2v+1)j\pi }{2V} \end{array}$$ (15)

Here, u=0,1,...,U−1, v=0,1,....,V−1 are two-dimensional signal sequences of U×V, the E(b) and E(j) are expressed as below,


$$\begin{array}{c} E\left(b\right)=\left\{ \begin{array}{c} @l@\frac{\text{1}}{\sqrt{\text{2}}},b=0\\ 1,b\neq 0 \end{array}\right. \end{array}$$ (16)

$$\begin{array}{c} E\left(j\right)=\left\{ \begin{array}{c} @l@\frac{\text{1}}{\sqrt{\text{2}}},j=0\\ 1,j\neq 0 \end{array}\right. \end{array}$$ (17)

Thus, the textural image $Tex_{\beta }$ is applied with DCT to get LL, LH, and HL band images. These band images are concatenated to get feature vector $K_{\beta }$.

### 3.5. Kidney stone detection in ultrasound kidney image with MFRANet

Kidney stone detection in ultrasound imaging involves identifying and characterizing renal calculi by determining their presence, size, shape, and anatomical location within kidneys or urinary tract. This process is performed using MFRANet, which integrates MFMANet [27] and GRA-Net [28] to capture complementary spatial and contextual feature information for accurate kidney stone detection. The extracted feature vector obtained from the descriptor computation stage is simultaneously provided as input to both MFMANet and GRA-Net, where MFMANet extracts multi-scale spatial and channel-aware features, while GRA-Net captures global contextual relationships using group response attention. Here, the Taylor concept enhances interpretability and the quality of extracted features with hybrid networks. The outputs generated from MFMANet and GRA-Net are integrated using the Taylor concept, where the weighted combination of both network responses produces a refined feature representation for final kidney stone classification. The extracted feature vector $K_{\beta }$ is given as input for both networks for detecting kidney stone. At first, MFMANet is implemented by multiplying weight $I_{\text{1}}$ with feature vector $K_{\beta }$ and accomplished output is $\Sigma I_{\text{1}}$. Next, multiply the resultant $\Sigma I_{\text{1}}$ by the MFMANet output afterward employing extracted feature $K_{\beta }$ as input, wherein final outcome achieved from MFMANet after multiplication is $J_{\text{1}}$. Afterwards, multiply weight $I_{\text{2}}$ by GRA-Net output after applying feature vector $K_{\beta }$ as input, and the output gained is again multiplied with $I_{\text{1}}$ to obtain the last resultant $J_{\text{2}}$. The fusion of MFMANet and GRA-Net enhances feature discrimination by integrating complementary multi-scale spatial, channel-aware, and global contextual information. This integrated feature representation enables MFRANet to more effectively distinguish renal calculi from surrounding tissues and ultrasound artifacts, thereby improving classification accuracy and reducing false detections during kidney stone identification. At the final stage, the Taylor concept is employed to achieve output $T_{\beta }$ by merging $J_{\text{1}}$ as well as $J_{\text{2}}$. The attained output $T_{\beta }$ may be under the stone or non-stone category. Fig 2 portrays the network architecture of MFRANet.

**Fig 2:**
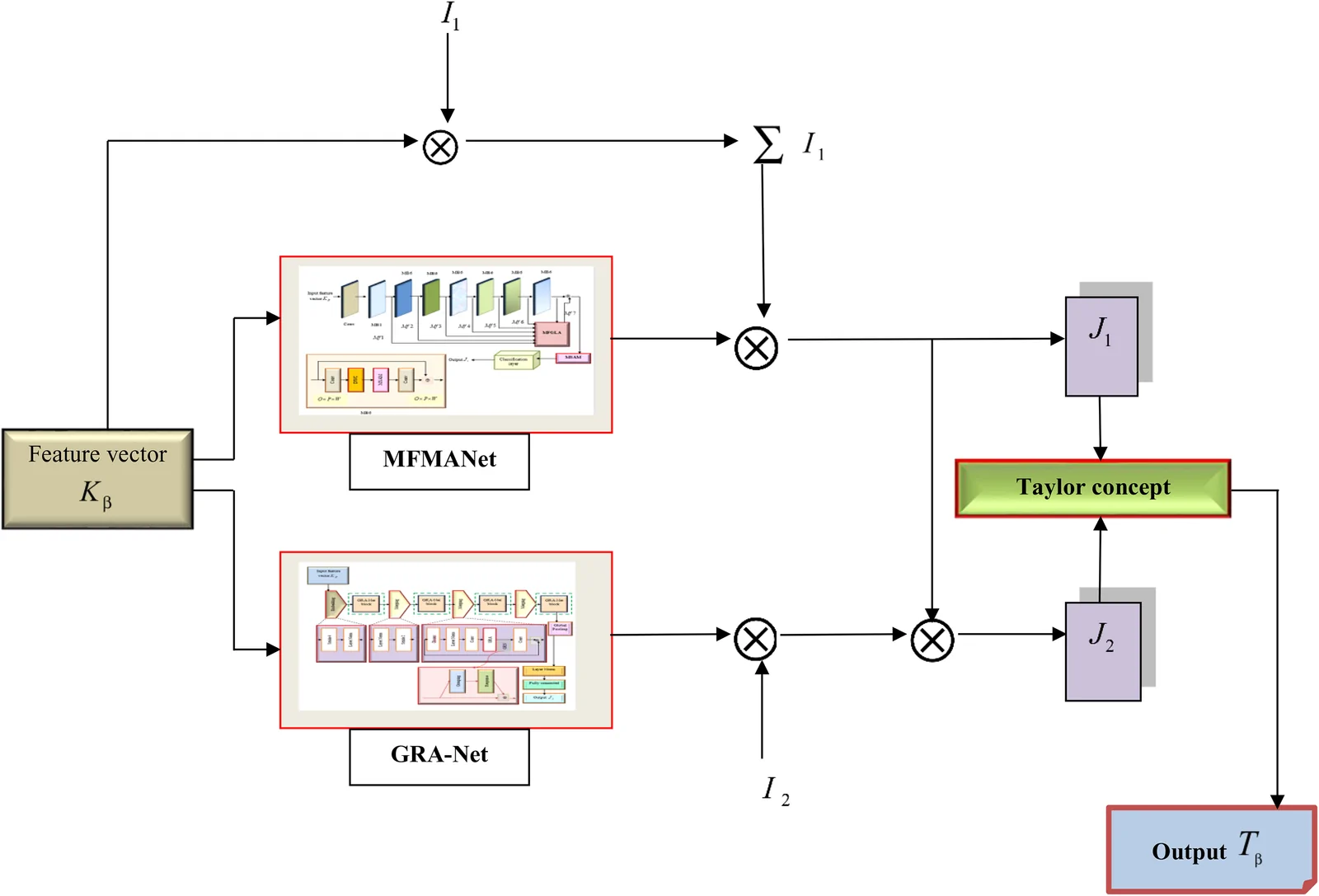
Schematic presentation of MFRANet.

#### 3.5.1. Architecture of MFMANet.

MFMANet is consists of 7 attention modules (MB) for extracting features, dual attention modules, as well as a convolutional classification layer. Every MB block performs deep extraction of features, while incorporating attention to enhance feature representation. Attention modules are designed for fusing multi-scale features and emphasize information most beneficial to model performance. Traditional Squeeze-and-Excitation (SE) mechanisms fuse features through global average pooling, but tend to ignore spatial details of feature maps, leading to potential information loss. Hence, the Multi-Scale Spatial Channel Attention Module (MSAM) is introduced, which captures spatial information while performing multi-scale channel attention. By combining spatial and channel feature extraction, MSAM enhances the model’s discriminative capability. In MB6 block, the feature map first undergoes 6-fold channel expansion, next Depth-Wise Convolution (DWC) to maintain the channel count. The output then passes through MSAM for extracting joint spatial and channel information. MB1 block maintains a constant count of channels, where L represents size of kernel utilized by DWC in MB block. Multi-scale feature extraction focuses on smaller pathological areas within the image. Features generated by seven MB blocks (Mf1 to Mf7) are then passed into the Multi-Feature Fusion Global–Local Attention (MFGLA) unit, which captures both local and global semantic information across multiple scales. Finally, the fused features are refined through additional MSAM before being classified by the final convolutional classification layer to generate Non-Small Cell Lung Cancer (NSCLC) subtype predictions. The feature vector $K_{\beta }$ is fed as input to MFMANet.

i) ***MSAM:*** Channel attention mechanism enhances important feature channels, while suppressing irrelevant or noisy ones. This structure enables the model to better distinguish among visually similar NSCLC subtypes in CT images. However, conventional channel attention focuses on inter-channel relationships and often neglects spatial feature information, which is crucial for accurately identifying small or localized pathological regions. Also, the Spatial Pyramid Pooling (SPP) component widely used in object detection networks captures information from multi-scale feature maps, preserving diverse spatial characteristics and improving task accuracy. The MSAM module is incorporated to enhance the representation of small kidney stones by emphasizing informative spatial regions and important feature channels. The multi-scale pooling operation enables the network to capture stone patterns with different sizes and appearances, while preserving local details that may be lost during conventional attention operations. Rather than using global average pooling, MSAM employs pooling at multiple spatial scales to preserve spatial details during channel-wise feature learning. The input feature maps undergo pooling at three different scales to extract spatial information. After Sigmoid activation, attention weights are applied to input feature map, emphasizing most important channels. This process yields an attention output that effectively preserves spatial information and enhances the discriminative power of the model. The channel attention mechanism, having multi-scale spatial information, is derived below,

$$\begin{array}{c} M_{o}=SA\left(\sum_{c\in scales}\frac{\text{1}}{c\times c}DW\left(PO\left(M_{i},c\right)\right)\right)⊗M_{i} \end{array}$$ (18)

Wherein, the input feature is $M_{i}$, pooling of scales is represented as PO, DWC is DW, Sigmoid activation function is SA, Element-wise multiplication is signified as ⊗, and the output feature is $M_{o}$.

ii) ***MFGLA:*** MFGLA blocks have dual key components. The MFGLA module integrates local and global contextual features from different MB blocks, improving the discrimination between stone and non-stone regions. MFF unit is responsible for extracting and integrating multi-scale information, where the Global–Local Attention unit captures both global and local contextual features via dual attention mechanisms. To effectively capture complex high-dimensional information, it is essential to learn multi-scale features from images. This enables more accurate pixel-level classification and supports the subtype categorization of medical images. Features mined from 7 MB blocks serve as input modules for further processing. Since the spatial dimensions of these feature maps differ, a down-sampling operation is first applied to align high-dimensional features with the spatial scale of low-dimensional ones. Subsequently, feature fusion is done using convolutional operations with channel expansion, where features are added and concatenated with channel dimension. Following this, an additional convolution layer is used to suppress redundant information in channel space and to reduce the cost of computation, resulting in refined and efficiently fused feature representations. The MFF is derived based on the formula. Features taken from seven MB blocks, Mf7 indicates the deepest feature utilizing $Mf_{d}\in N^{O_{d}\times P_{d}\times W_{d}},d\in \left(1,2,...,7\right)$.

$$\begin{array}{c} Mf_{fd}=Tf_{fd}^{T}Mf_{d},d=1,2,....,7 \end{array}$$ (19)

$$\begin{array}{c} Mf_{sd}=Tf_{q(d-1)}^{T}Mf_{f(d-1)}⊕Mf_{fd},d=1,2,....,7 \end{array}$$ (20)

$$\begin{array}{c} Mf_{s1}=Tf_{q1}^{T}Mf_{f1} \end{array}$$ (21)

Herein, down-sampling operation on feature $Mf_{d}$ to attain $Mf_{fd}$ is $Tf_{fd}^{T}Mf_{d}$, convolution of channel expansion employed on $Mf_{fd}$ to attain a similar size as next level $Mf_{f(d+1)}$ is $Tf_{qd}^{T}Mf_{fd}$ and then added to it for obtaining $Mf_{sd}$, dual element-wise summation features is ⊕, and further the resultant of MFF module is derived as below,


$$\begin{array}{c} M_{o}=Tf_{gd}^{T}Con\left(\left[Mf_{s1},Mf_{s2},.....,Mf_{s7}\right]\right) \end{array}$$ (22)

Herein, concatenation of features is Con(.), and convolution of channel compression for achieving the module resultant aggregated feature $M_{o}$ is $Tf_{gd}^{T}$. A single attention mechanism typically focuses on either global or local information, but not both simultaneously. Hence, the MFGLA structure is introduced with dual branches. The global attention branch applies global average pooling to the aggregated feature $M_{o}$ obtained from the MFF module, extracting comprehensive global contextual information. The local attention branch employs convolutional processes to capture fine-grained local feature details. The computational process is described as follows,


$$\begin{array}{c} G_{s}=PW\left(R_{e}\left(B_{n}\left(PW\left(G_{ap}\left(M_{o}\right)\right)\right)\right)\right) \end{array}$$ (23)

$$\begin{array}{c} L_{s}=PW\left(R_{e}\left(B_{n}\left(PW\left(K_{\beta }\right)\right)\right)\right) \end{array}$$ (24)

$$\begin{array}{c} J_{\text{1}}=R_{e}\left(B_{n}\left(PW\left(SA\left(G_{s}⊕L_{s}\right)\right)\right)\right)*\sum K_{\beta }*I_{\text{1}} \end{array}$$ (25)

Here, point-wise conclusion is PW(.), global attention branch is G(.), another local attention branch is L(.), global average pooling is $G_{ap}(.)$, batch normalization is $B_{n}(.)$, ReLu function is $R_{e}(.)$, MFMANet output is $J_{\text{1}}$, as well as sigmoid activation function is SA(.). Thus, substituting Eq. (24) in Eq. (25), it becomes,


$$\begin{array}{c} J_{\text{1}}=R_{e}\left(B_{n}\left(PW\left(SA\left(G_{s}⊕\left(PW\left(R_{e}\left(B_{n}\left(PW\left(K_{\beta }\right)\right)\right)\right)\right)\right)\right)\right)\right)*\sum K_{\beta }*I_{\text{1}} \end{array}$$ (26)

The multi-scale attention mechanism enables MFMANet to detect kidney stones with varying sizes and appearances by preserving important feature details, particularly for small stones with low contrast. Fig 3 portrays the architecture of MFMANet and Table 1 displays Layer-wise configuration of MFMANet.

**Table 1:** Layer-wise configuration of MFMANet.

Layer	Kernel	Stride	Activation	Input Shape	Output Shape
Conv1	3 × 3, 32 filters	2	GELU	224 × 224 × 3	112 × 112 × 32
MB1	DWConv 3 × 3	1	GELU	112 × 112 × 32	112 × 112 × 32
MB2	DWConv 3 × 3	2	GELU	112 × 112 × 32	56 × 56 × 64
MB3	DWConv 3 × 3	2	GELU	56 × 56 × 64	28 × 28 × 128
MB4	DWConv 3 × 3	2	GELU	28 × 28 × 128	14 × 14 × 256
MB5	DWConv 3 × 3	1	GELU	14 × 14 × 256	14 × 14 × 256
MB6	1 × 1 Expansion (×6) → DWConv 3 × 3 → MSAM → 1 × 1 Projection	2	GELU	14 × 14 × 256	7 × 7 × 512
MB7	DWConv 3 × 3	1	GELU	7 × 7 × 512	7 × 7 × 512
MFGLA	1 × 1 Conv + Global/Local Attention	1	GELU	7 × 7 × 512	7 × 7 × 512
MSAM	Multi-scale Attention	—	Sigmoid	7 × 7 × 512	7 × 7 × 512
Global Average Pooling	Global average pooling	—	—	7 × 7 × 512	1 × 1 × 512

**Fig 3:**
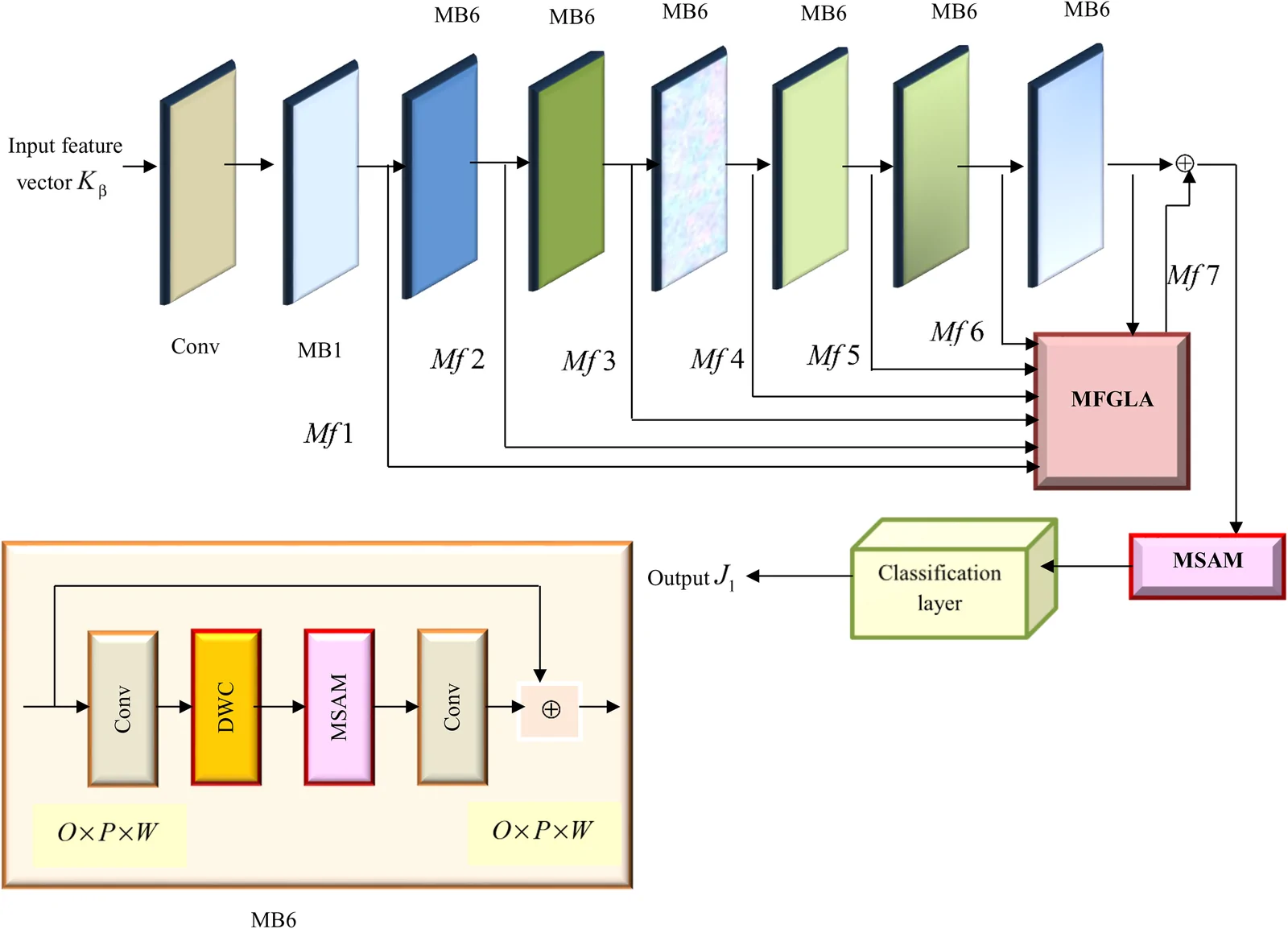
Structure of MFMANet.

#### 3.5.2. Structure of GRA-Net.

GRA-Net is a DCNN architecture that enhances feature representation by combining global receptive field expansion with attention mechanisms to capture both contextual and local image details. In ultrasound images, kidney stones appear as small, low-contrast regions affected by speckle noise and surrounding tissues. Group response attention improves feature discrimination by emphasizing stone-related patterns while suppressing irrelevant information, thereby enhancing detection robustness. GRA-Net models of varying dimensions are constructed by adjusting the network’s width and depth through the stacking of unit blocks. Embedding, Merging, and the GRA-Net Block of GRA-Net are elucidated below. The feature vector $K_{\beta }$ is given to GRA-Net as input.

i) ***Group response attention:*** Rather than utilizing element-wise activations, the attention mechanisms are leveraged, facilitating information sharing among neurons to jointly determine their responses. For input feature vector $K_{\beta }$, the group response attention comprises feature grouping, feature attention, and group response.
  a) ***Feature grouping:*** Features are divided into groups and consider feature vector $K_{\beta }$ with dimensions X,Y,Z, where X,Y,Z are height, width, and channel of features. Here, spatial dimensions are divided into groups.
  b) ***Group response:*** Response function $R_{f}(.)$ is applied to every group, and the corresponding equation is given below,

$$\begin{array}{c} R_{f}\left(K_{\beta_{\left(s,t,u\right)}}\right)=\frac{{\left(K_{\beta_{\left(s,t,u\right)}}\right)}^{\text{2}}}{\xi +\frac{\text{1}}{\omega }.\sum_{\upsilon =1,...,\omega }{\left(K_{\beta_{\left(s,t,\upsilon \right)}}\right)}^{\text{2}}}\in \mathbb{Z}\mathbb{C} \end{array}$$ (27)

Herein, s,t,u represents dimensions height, width, and channel of feature, ξ represents a small float added to the denominator, and ω indicates the number of elements. Consider feature value distance relative to zero. The absolute function $\left|K_{\beta_{\left(s,t,u\right)}}\right|$ is applied, and its performance remains less than the square function. Next, mean square is used as the denominator to calculate the response value for every group element. If ${\left(K_{\beta_{\left(s,t,u\right)}}\right)}^{\text{2}}$ is more than the mean, the response value $R_{f}\left(K_{\beta_{\left(s,t,u\right)}}\right)$ is more than 1. Also, when ${\left(K_{\beta_{\left(s,t,u\right)}}\right)}^{\text{2}}$ is less than the mean, the $R_{f}\left(K_{\beta_{\left(s,t,u\right)}}\right)$ remains less than 1. The weights are shifted from lower energy members to higher energy members. Here, connections amongst the elements are introduced. Every response value $R_{f}\left(K_{\beta_{\left(s,t,u\right)}}\right)$ is jointly found by group members.

c) ***Feature attention:*** Group response values are considered as scaling feature coefficients. The formula determining feature attention is derived below,

$$\begin{array}{c} T\left(K_{\beta }\right)=R_{f}\left(K_{\beta }\right).K_{\beta }\in {\mathbb{Z}\mathbb{C}}^{X\times Y\times Z} \end{array}$$ (28)

Dual learnable parameters $\sigma \in {\mathbb{Z}\mathbb{C}}^{1\times 1\times Z}$ and $\varsigma \in {\mathbb{Z}\mathbb{C}}^{1\times 1\times Z}$ are added for simplification,


$$\begin{array}{c} T\left(K_{\beta }\right)=\left(\sigma .R_{f}\left(K_{\beta }\right)+1\right).K_{\beta }+\varsigma \in {\mathbb{Z}\mathbb{C}}^{X\times Y\times Z} \end{array}$$ (29)

Herein, σ and ς are initialized to zero. For larger-size models, the above equation is modified as,


$$\begin{array}{c} T\left(K_{\beta }\right)=\left(\sigma .{log}_{\text{2}}\left(1+R_{f}\left(K_{\beta }\right)\right)+1\right).K_{\beta }+\varsigma \in {\mathbb{Z}\mathbb{C}}^{X\times Y\times Z} \end{array}$$ (30)

Also, ${log}_{\text{2}}\left(1+R_{f}\left(K_{\beta }\right)\right)=0$ when $R_{f}\left(K_{\beta }\right)=0$ and ${log}_{\text{2}}\left(1+R_{f}\left(K_{\beta }\right)\right)=1$ when $R_{f}\left(K_{\beta }\right)=1$.

ii) ***GRA block:*** This mechanism is applied within each unit block. Normalization serves as a crucial component, enhancing model convergence and mitigating overfitting. An inverted bottleneck structure from the Transformer block is adopted, where the hidden dimension of the MLP is expanded to four times that of the input size. GRA-Net replaces the conventional activation function within the MLP with the GRA mechanism. Consequently, traditional activation functions are entirely omitted from the network. The resulting GRA block maintains a straightforward structure that is both conceptually simple and computationally efficient to implement. The integration of global and local contextual features enables GRA-Net to capture stone-specific details and surrounding kidney information that improves the discrimination between kidney stones and normal tissues while reducing false detections caused by ultrasound artifacts.
iii) ***GRA-Net:*** This is generated by stacking unit blocks and is expressed as below with sets of channel size $C_{s}$ and count of blocks $C_{b}$.

$$\begin{array}{c} C_{s}=\left(c_{s1},c_{s2},c_{s3},c_{s4}\right),C_{b}=\left(c_{b1},c_{b2},c_{b3},c_{b4}\right) \end{array}$$ (31)

The general equation of the GRA-Net block is indicated as below,


$$\begin{array}{c} J_{\text{2}}=\rho \left(K_{\beta }.I+B_{ia}\right)*I_{\text{2}}*J_{\text{1}} \end{array}$$ (32)

Here, $J_{\text{2}}$ is the output attained from GRA-Net, I specifies weight, $B_{ia}$ indicates weight, and $J_{\text{1}}$ is output from MFMANet. Thus, Eq. (32) becomes, after substituting Eq. (26), as given below,


$$\begin{array}{c} J_{\text{2}}=\rho \left(K_{\beta }.I+B_{ia}\right)*I_{\text{2}}*\left[R_{e}\left(B_{n}\left(PW\left(SA\left(G_{s}⊕\left(PW\left(R_{e}\left(B_{n}\left(PW\left(K_{\beta }\right)\right)\right)\right)\right)\right)\right)\right)\right)*\sum K_{\beta }*I_{\text{1}}\right] \end{array}$$ (33)

Fig 4 portrays the architecture of GRA-Net and Table 2 illustrates Layer-wise configuration of GRA-Net.

**Table 2:** Layer-wise configuration of GRA-Net.

Layer/Block	Kernel/Operation	Stride	Activation	Input Shape	Output Shape
Embedding Conv	3 × 3, 64 filters	2	GELU	224 × 224 × 3	112 × 112 × 64
LayerNorm	Layer Normalization	—	—	112 × 112 × 64	112 × 112 × 64
GRA Block ×2	DWConv 3 × 3	1	GELU	112 × 112 × 64	112 × 112 × 64
Merge1	Conv 3 × 3	2	GELU	112 × 112 × 64	56 × 56 × 128
LayerNorm	Layer Normalization	—	—	56 × 56 × 128	56 × 56 × 128
GRA Block ×2	DWConv 3 × 3	1	GELU	56 × 56 × 128	56 × 56 × 128
Merge2	Conv 3 × 3	2	GELU	56 × 56 × 128	28 × 28 × 256
LayerNorm	Layer Normalization	—	—	28 × 28 × 256	28 × 28 × 256
GRA Block ×4	DWConv 3 × 3	1	GELU	28 × 28 × 256	28 × 28 × 256
Merge3	Conv 3 × 3	2	GELU	28 × 28 × 256	14 × 14 × 512
LayerNorm	Layer Normalization	—	—	14 × 14 × 512	14 × 14 × 512
GRA Block ×2	DWConv 3 × 3	1	GELU	14 × 14 × 512	14 × 14 × 512
Global Average Pooling	Global Average Pooling	—	—	14 × 14 × 512	1 × 1 × 512
Fully Connected	Fully Connected	—	Softmax	512	2

**Fig 4:**
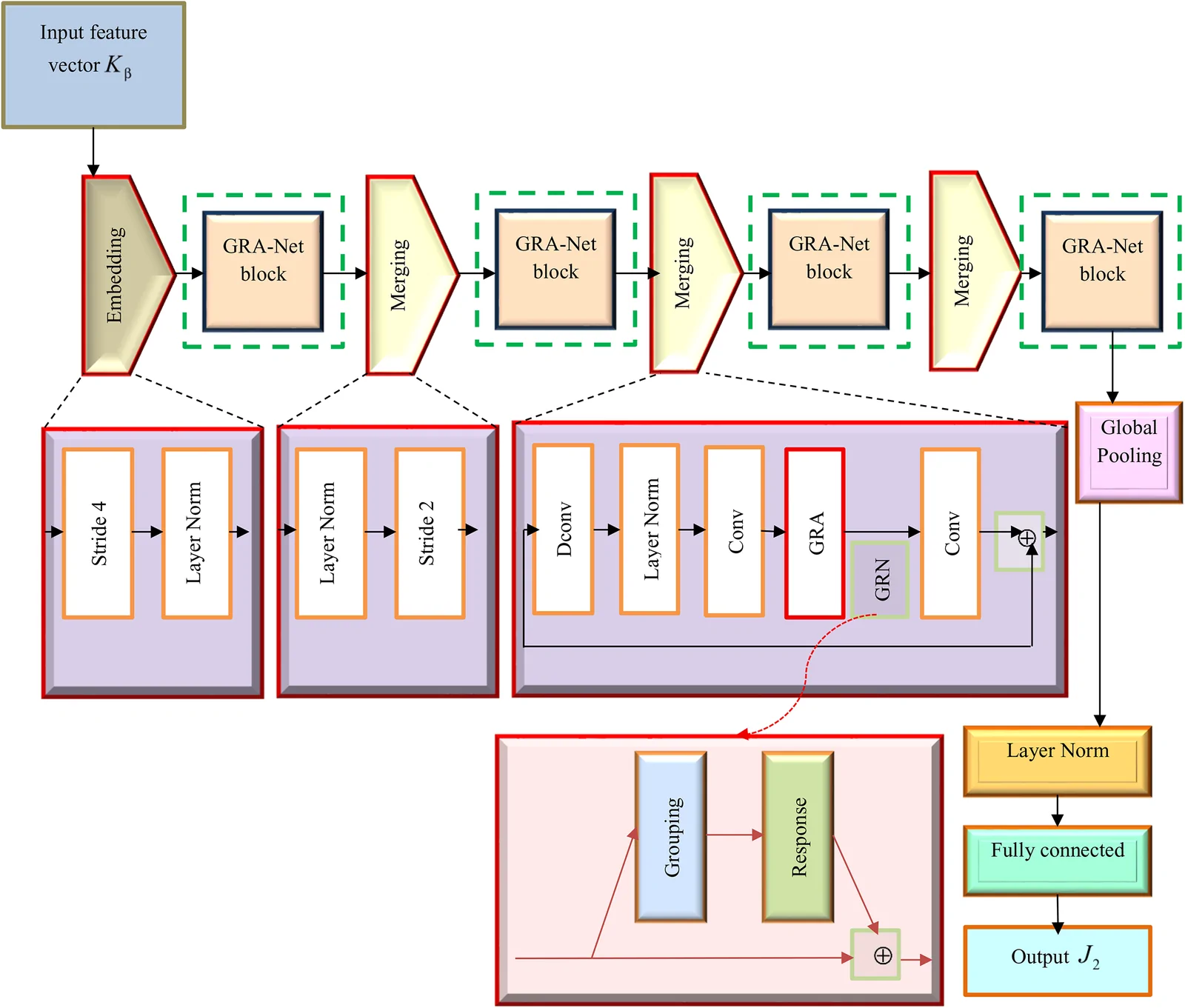
Architecture of GRA-Net.

#### 3.5.3. Taylor concept.

Taylor concept [30] provides a mathematical representation of a function as an infinite series, where each term is derived from the values of the function’s derivatives at a specific point. The Taylor-based fusion strategy combines complementary features from MFMANet and GRA-Net by modeling their interactions beyond conventional addition or concatenation. It integrates multi-scale spatial and contextual features to improve discrimination between stone and non-stone regions. The general expression of the Taylor series is given below,


$$\begin{array}{c} z(j+1)=z\left(j\right)+\frac{z^{\prime }\left(j\right)}{1!} \end{array}$$ (34)

$$\begin{array}{c} z^{\prime }\left(j\right)=\frac{z\left(j\right)-z(j-\text{ƛ})}{\text{ƛ}} \end{array}$$ (35)

Assume, ƛ=1 and substitute $z^{\prime }\left(j\right)$ in Eq. (34),


$$\begin{array}{c} z(j+1)=z\left(j\right)+\frac{z\left(j\right)-z(j-1)}{1!} \end{array}$$ (36)

$$\begin{array}{c} z(j+1)=2.z\left(j\right)-z(j-1) \end{array}$$ (37)

Let, $z(j+1)=T_{\beta },z\left(j\right)=J_{\text{1}},z(j-1)=J_{\text{2}}$,


$$\begin{array}{c} T_{\beta }=2J_{\text{1}}-J_{\text{2}} \end{array}$$ (38)

$$\begin{array}{c} \begin{array}{c} T_{\beta }=2\left[R_{e}\left(B_{n}\left(PW\left(SA\left(G_{s}⊕\left(PW\left(R_{e}\left(B_{n}\left(PW\left(K_{\beta }\right)\right)\right)\right)\right)\right)\right)\right)\right)*\sum K_{\beta }*I_{\text{1}}\right]-\\ \left[\rho \left(K_{\beta }.I+B_{ia}\right)*I_{\text{2}}*\left[R_{e}\left(B_{n}\left(PW\left(SA\left(G_{s}⊕\left(PW\left(R_{e}\left(B_{n}\left(PW\left(K_{\beta }\right)\right)\right)\right)\right)\right)\right)\right)\right)*\sum K_{\beta }*I_{\text{1}}\right]\right] \end{array} \end{array}$$ (39)

$$\begin{array}{c} T_{\beta }=\left[R_{e}\left(B_{n}\left(PW\left(SA\left(G_{s}⊕\left(PW\left(R_{e}\left(B_{n}\left(PW\left(K_{\beta }\right)\right)\right)\right)\right)\right)\right)\right)\right)*\sum K_{\beta }*I_{\text{1}}\right]\left[2-\rho \left(K_{\beta }.I+B_{ia}\right)*I_{\text{2}}\right] \end{array}$$ (40)

Here, $I,I_{\text{1}},I_{\text{2}}$ are weights, $T_{\beta }$ is resultant attained for kidney stone detection, where the output indicates stone or non-stone. Algorithm 1 is pseudocode of MFRANet for kidney stone diagnosis.

**Algorithm 1.** Pseudocode of MFRANet model

**Input**

 **Training**: Training feature $K_{\beta_{tr}}$, weight of MFMANet $I_{\text{1}}$, Weight of GRA-Net $I_{\text{2}}$, and training data labels

 **Testing:** Testing feature $K_{\beta_{te}}$, weight of MFMANet $I_{\text{1}}$, Weight of GRA-Net $I_{\text{2}}$, and testing data labels

**Output**

 Weight and bias of MFRANet

 Predicted labels of MFRANet $T_{\beta }$

**Required parameters**

 Maximal Epoch Eph

 Learning rate Lre

 Batch size

**Start**

**Initialization**

Weight and bias of MFRANet randomly

Initialize batch size, epoch, and learning rate

**//Training phase**

 **while** j<Eph

  for training feature $K_{\beta_{tr}}$

  Implement MFMANet by multiplying weight $I_{\text{1}}$ with extracted feature $K_{\beta }$ and achieved outcome to be $\Sigma I_{\text{1}}$

  Multiply output $\Sigma I_{\text{1}}$ with MFMANet output after application of extracted feature $K_{\beta }$ as input; the final resultant gained from MFMANet after multiplication is $J_{\text{1}}$.

  $J_{\text{1}}=R_{e}\left(B_{n}\left(PW\left(SA\left(G_{s}⊕\left(PW\left(R_{e}\left(B_{n}\left(PW\left(K_{\beta }\right)\right)\right)\right)\right)\right)\right)\right)\right)*\sum K_{\beta }*I_{\text{1}}$

  Multiply weight $I_{\text{2}}$ with GRA-Net output after application of extracted feature $K_{\beta }$ as input, and the attained output is to be again multiplied with $I_{\text{1}}$ to get the final resultant $J_{\text{2}}$.

  $J_{\text{2}}=\rho \left(K_{\beta }.I+B_{ia}\right)*I_{\text{2}}*\left[R_{e}\left(B_{n}\left(PW\left(SA\left(G_{s}⊕\left(PW\left(R_{e}\left(B_{n}\left(PW\left(K_{\beta }\right)\right)\right)\right)\right)\right)\right)\right)\right)*\sum K_{\beta }*I_{\text{1}}\right]$

  Apply Taylor concept to achieve output $T_{\beta }$ by merging $J_{\text{1}}$ and $J_{\text{2}}$.

  $T_{\beta }=\left[R_{e}\left(B_{n}\left(PW\left(SA\left(G_{s}⊕\left(PW\left(R_{e}\left(B_{n}\left(PW\left(K_{\beta }\right)\right)\right)\right)\right)\right)\right)\right)\right)*\sum K_{\beta }*I_{\text{1}}\right]\left[2-\rho \left(K_{\beta }.I+B_{ia}\right)*I_{\text{2}}\right]$

**end for**

 Find error err by considering $T_{\beta }$ and training feature

 Update bias and weight of MFRANet based on Adam optimizer

  j++

 **End while**

**For testing phase**

 For every testing feature $K_{\beta_{te}}$

  Estimate $T_{\beta }$, $J_{\text{1}}$ and $J_{\text{2}}$ with tuned MFRANet

  Estimate predicted label $T_{\beta }$

 **End for**

End

Thus, the attained output is $T_{\beta }$, from which stone or non-stone output is predicted. From this, the stone output is again processed for the segmentation process to find its measurement.

### 3.6. Kidney stone segmentation using O-DAT

After kidney stone detection, segmentation isolates the stone region for accurate shape and size measurement. O-DAT improves boundary localization by combining convolutional features, Transformer-based context, and attention mechanisms, enabling precise stone extraction and reliable size estimation. Kidney stone Segmentation refers to segmenting the stone for measuring its size and shape. This is done by O-DAT [29], which is an advanced DL framework. Within the skip connections, the ODA-Blocks act to suppress redundant information and sharpen reconstruction. The kidney region segmented output $S_{\beta }$ and kidney stone detected output $T_{\beta }$ are together indicated as $ST_{\beta }$, such that it is indicated as below,


$$\begin{array}{c} ST_{\beta }=\left\{S_{\beta },T_{\beta }\right\} \end{array}$$ (41)

This $ST_{\beta }$ is allowed as input for the kidney stone segmentation process.

#### 3.6.1. ODA-Block.

The ODA-Block effectively extracts features from both channel and spatial dimensions of an image, facilitating a more precise and nuanced representation of image characteristics. By integrating this block into the encoder and skip connections, this model captures intricate details and suppresses redundant information, thereby enhancing the segmentation performance. This dual-dimensional feature extraction is crucial for accurately delineating structures within medical images, ensuring that relevant features are preserved while irrelevant information is minimized. This block integrates Channel Attention Module (CAM) and Spatial Attention Module (SAM).

i) ***SAM:*** This is better at capturing spatial interdependencies over many positions within feature maps via weighted aggregation. The feature similarities help in assigning the weights at various locations. First, SAM acquires a local feature tensor $B\in E^{\Gamma \times H\times I}$, where this tensor is processed via a series of nonlinear and convolutional processes. Here, the count of channels is specified as Γ, height and width are H and I. This results in three new feature tensors, such as K,Γ, and M, where K, and Γ are reshaped to $E^{\Gamma \times \Lambda }$, with Λ=H×I. Here, the overall number of pixels is signified as Λ. Between the transpose of Γ and K, the matrix multiplication is done, which is followed by the softmax procedure to form the spatial attention matrix $Z_{sp}$. Thus, the output of SAM is derived as below,

$$\begin{array}{c} N_{s}=ℑ\left(\sum_{p=1}^{\Lambda }Z_{sp}.\varepsilon \left(M_{p}\right)\right)+B_{s} \end{array}$$ (42)

Herein, ε is a nonlinear function applied to features $M_{p}$ prior weighted sum, and ℑ indicates a learnable parameter controlling spatial attention influence.

ii) ***CAM:*** This is designed for excelling channel-wise extraction of features. CAM reshapes the input feature $B\in E^{\Gamma \times H\times I}$ directly to $E^{\Gamma \times \Lambda }$ performing matrix multiplication amongst B and its transpose. Further, a softmax layer is employed for gaining the channel attention matrix $C\in E^{\Gamma \times \Gamma }$. Then, the resultant of CAM is designed as below,

$$\begin{array}{c} N_{s}=ℜ\left(\sum_{p=1}^{\Lambda }\Upsilon_{sp}.\gamma \left(B_{p}\right)\right)+B_{s} \end{array}$$ (43)

Min-max scaling operation is γ applied to which regularizes feature scales $B_{p}$, and ℜ is learnable parameter dictating extent of channel attention’s influence.

iii) ***ODA:*** In ODA, two components are involved, one dominated by SAM and another with CAM. The first component processes input features by applying a convolution reducing channel count, producing ${ℑ}_{\text{1}}$ to simplify feature extraction for SAM. After SAM performs extraction of feature, the other convolution is applied to generate ${\mathrm{\backslash lower}0.5em\mathrm{\backslash smash}⌢ℑ}_{\text{1}}$. Similarly, by the second process, the ${\mathrm{\backslash lower}0.5em\mathrm{\backslash smash}⌢ℑ}_{\text{2}}$ is formed. After extraction of \lower0.5em\smash⌢ℑ1 and ${\mathrm{\backslash lower}0.5em\mathrm{\backslash smash}⌢ℑ}_{\text{2}}$, the resultant from ODA-Block is given as below,

$$\begin{array}{c} T=Conv\left(\phi \left({\overset{\mathrm{\backslash lower}0.5em\mathrm{\backslash smash}⌢}{\}}ℑ}_{\text{1}}+{\overset{\mathrm{\backslash lower}0.5em\mathrm{\backslash smash}⌢}{\}}ℑ}_{\text{2}}\right)\right) \end{array}$$ (44)

Herein, ϕ specifies the sigmoid function.

#### 3.6.2. Encoder.

This layer merges components, such as convolutional blocks, embedding layers, transformer layers, and ODA blocks. ODA modules, placed before the Transformer, refine features by attention mechanisms to emphasize local spatial and channel-level details. Moreover, the embedding layer adapts feature dimensions for the Transformer, enabling a seamless blend of local and global representations. This synergy significantly improves the model’s ability to capture fine anatomical details in medical images.

#### 3.6.3. Skip connections.

These connections bridge the semantic gap between the decoder and encoder. Moreover, ODA-blocks are incorporated within every three skip connection layers. Integrating ODA-Blocks within skip connections enables more effective refinement of sparsely encoded features from channel and positional perspectives. This approach helps in extracting more informative representations while minimizing redundancy.

#### 3.6.4. Decoder.

The decoder comprises a segmentation head, feature concatenation, and patch expansion layers for upsampling. Feature integration combines skip connection feature maps with prevailing ones, which is essential for appropriate reconstruction. The segmentation head resizes the final resultant feature map back to its original size. Patch expansion layers progressively double the resolution of the input feature map at every stage, restoring the resolution of the image. The procedure begins with a convolutional layer process, followed by upsampling to enhance the feature maps. Stone segmented output attained from O-DAT is $O_{\beta }$. Fig 5 portrays the architecture of O-DAT.

**Fig 5:**
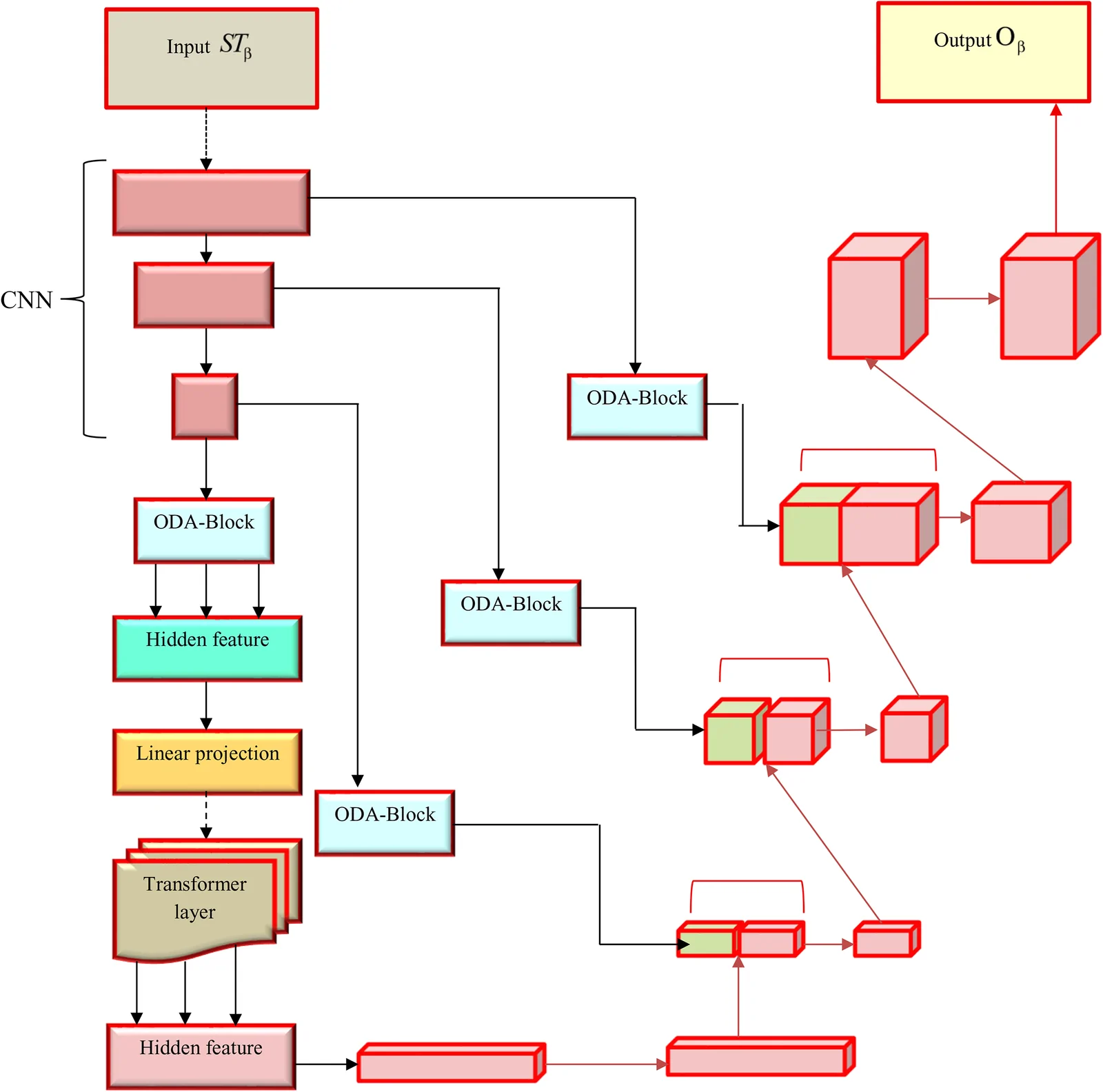
Architecture of O-DAT.

### 3.7. Kidney stone size measurement from segmented stone image

After detecting and segmenting the kidney stone, its size is measured in millimetres (mm). This helps to analyse the stone in detail to give an appropriate treatment. The measurement is done based on the formula below,


$$\begin{array}{c} St_{mm}=St_{pix}\times \Omega \end{array}$$ (45)

Herein, $St_{mm}$ indicates stone size in mm, $St_{pix}$ indicates stone size in pixels, and Ω is pixel to mm factor. The same conversion factor is applied consistently to all ultrasound images to obtain the corresponding stone-size measurement. The resulting value represents the image-based estimated stone size. Finally, the stone size is measured in mm for accurate and effective treatment.

## 4. Results and discussions

MFRANet for kidney stone detection is assessed for its efficiency, and results are discussed in this section.

### 4.1. Experimental setup

The proposed MFRANet is executed using the Python tool. MFRANet’s network parameter are given in Table 3.

**Table 3:** Network parameters.

Parameters	Values
Optimizer	Adam
Learning rate	0.001
Loss	Categorical cross entropy
Weight decay	1e-5
Epochs	100
Dropout	0.3
Batch size	64
Activation	GELU
Number of attention heads	5

### 4.2. Dataset description

The datasets used are kidney-stone-ultrasound Computer Vision Model dataset [20], KSSD2025- Kidney Stone Segmentation Dataset [21], and CT Kidney Dataset: Normal-Cyst-Tumor and Stone [22]. Here, KSSD2025- Kidney Stone Segmentation Dataset and CT Kidney Dataset: Normal-Cyst-Tumor and Stone are together considered in the second case.

#### 4.2.1. Kidney-stone-ultrasound Computer Vision Model dataset.

This dataset consists of 5431 images, that is split into train set with 4942 images, valid set with 260 images, and test set with 229 images [20]. This is an object detection dataset and model hosted on Roboflow under the project kidney-stone-ultrasound. The model is deployable via Application Programming Interface (API) or on‐device, like Docker, Jetson, Raspberry Pi, web, iOS using Roboflow’s inference ecosystem. Dataset was divided into three subsets: 80% for training, 10% for validation, and 10% for testing. Dataset split was performed at the image level.

After preprocessing, the number of stone and non-stone images included in the training, validation, and testing sets for Kidney-stone-ultrasound Computer Vision Model dataset are given in Table 4.

**Table 4:** Number of stone and non-stone images after processing for the Kidney-stone-ultrasound Computer Vision Model dataset.

Split	Stone	Normal	Total
Training (80%)	2485	1,860	4,345
Validation (10%)	311	233	544
Testing (10%)	310	232	542
Total	3106	2325	5431

#### 4.2.2. KSSD2025-kidney stone segmentation dataset.

KSSD2025 is a specialized dataset focused exclusively on kidney stone cases, providing precise ground-truth masks to support the development and benchmarking of AI-based segmentation models. From the initial 12,446 images, 838 were selected for manual annotation based on the presence of stones and the axial orientation, which offers superior anatomical context for segmentation tasks [21]. From this dataset, the kidney stone images or abnormal images are taken for analysis. Dataset was allocated into training, validation, and testing sets with proportions of 80%, 10%, and 10%. The dataset split was performed at the image level.

The ultrasound and CT datasets possess different imaging characteristics. Ultrasound images are affected by speckle noise and low contrast, whereas CT images provide clearer anatomical information. Therefore, the datasets are processed according to their modality characteristics, and evaluation is performed separately for reliable assessment.

Table 5 summarizes the number of stone and non-stone images in training, validation, and testing sets for KSSD2025 and CT Kidney Dataset after preprocessing.

**Table 5:** Number of stone and non-stone images after processing for KSSD2025 and CT kidney dataset.

Split	Stone	Normal	Total
Training (80%)	670	4062	4732
Validation (10%)	84	508	592
Testing (10%)	84	507	591
Total	838	5077	5915

#### 4.2.3. CT kidney dataset: normal-cyst-tumour and stone.

This dataset [22] was collected from PACS of several hospitals in Bangladesh, where patients had been previously diagnosed with kidney-related conditions, including tumours, cysts, stones, or normal findings. Each DICOM study was carefully reviewed and categorized according to a single diagnosis, from which batches of region-of-interest DICOM images were created for each radiological finding. The final dataset comprises 12,446 unique images, distributed as follows: 5077 normal, 3709 cyst, 2283 tumour, and 1377 stone cases. From this dataset, the normal kidney images are taken for analysis.

### 4.3. Evaluation metrics

Metrics for evaluation are accuracy and TPR, which are elaborated below.

#### 4.3.1. Accuracy.

Accuracy measures the overall performance of the model and indicates its ability to correctly detect kidney stones [31], that is represented as below.


$$\begin{array}{c} Acc_{yy}=\frac{F^{*p}+F^{*n}}{F^{*p}+F^{*n}+M^{*p}+M^{*n}} \end{array}$$ (46)

Herein, $Acc_{yy}$ indicates accuracy, true negative is $F^{*n}$, true positive is $F^{*p}$, false negative is $M^{*n}$, and false positive is $M^{*p}$.

#### 4.3.2. Precision.

Precision [32] is defined as the percentage of correctly predicted kidney-stone cases among the total cases predicted as positive (stone) by the classification model.


$$\begin{array}{c} precision=\frac{F^{*p}}{F^{*p}+M^{*p}} \end{array}$$ (47)

#### 4.3.3. F1-score.

F1-score [32] measures the balance between the precision and TPR of the model in correctly detecting kidney stone cases.


$$\begin{array}{c} F1-score=\frac{2\times F^{*p}}{2\times F^{*p}+M^{*p}+M^{*n}} \end{array}$$ (48)

#### 4.3.4. TPR.

TPR [31] represents the proportion of actual positive cases of kidney stones that are correctly identified by the model.


$$\begin{array}{c} T_{prr}=\frac{F^{*p}}{F^{*p}+M^{*n}} \end{array}$$ (49)

TPR is signified by $T_{prr}$.

### 4.4. Experimental outcomes

Experimental results of kidney stone detection by MFRANet from the Kidney-stone-ultrasound Computer Vision Model dataset with stone measurement are given in Fig 6. Fig 6a) represents input image-1, Fig 6b) indicates input image-2, Fig 6c) specifies denoised image-1, Fig 6d) portrays denoised image-2, Fig 6e) is kidney region segmented image-1, Fig 6f) describes kidney region segmented image-2, Fig 6g) enumerates rBRIEF descriptor applied image-1, Fig 6h) is rBRIEF descriptor applied image-2, Fig 6i) is output image-1, Fig 6j) illustrates output image-2, Fig 6k) indicates stone segmented image, and Fig 6l) represents kidney stone with size.

**Fig 6:**
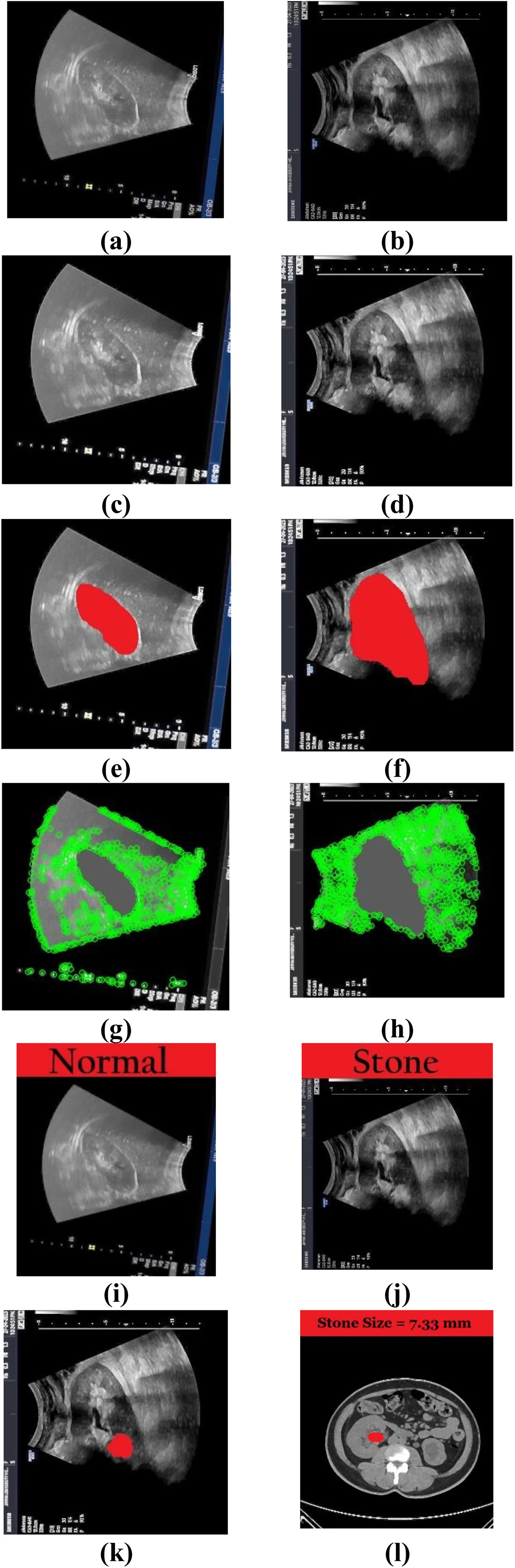
Experimental results of kidney stone detection from Kidney-stone-ultrasound Computer Vision Model dataset, a) input image-1, b) input image-2, c) denoised image-1, d) denoised image-2, e) kidney region segmented image-1, f) kidney region segmented image-2, g) rBRIEF descriptor applied image-1, h) rBRIEF descriptor applied image-2, i) output image-1, j) output image-2, k) stone segmented image, l) kidney stone with size.

Experimental outcomes of detecting kidney stones by MFRANet from KSSD2025 and the CT Kidney Dataset with stone measurement are given in Fig 7. Fig 7a) resembles input image-1, Fig 7b) indicates input image-2, Fig 7c) illustrates denoised image-1, Fig 7d) represents denoised image-2, Fig 7e) portrays kidney region segmented image-1, Fig 7f) describes kidney region segmented image-2, Fig 7g) signifies rBRIEF descriptor applied image-1, Fig 7h) is rBRIEF descriptor applied image-2, Fig 7i) represents output image-1, Fig 7j) illustrates output image-2, Fig 7k) indicates stone segmented image, and Fig 7l) shows kidney stone with size.

**Fig 7:**
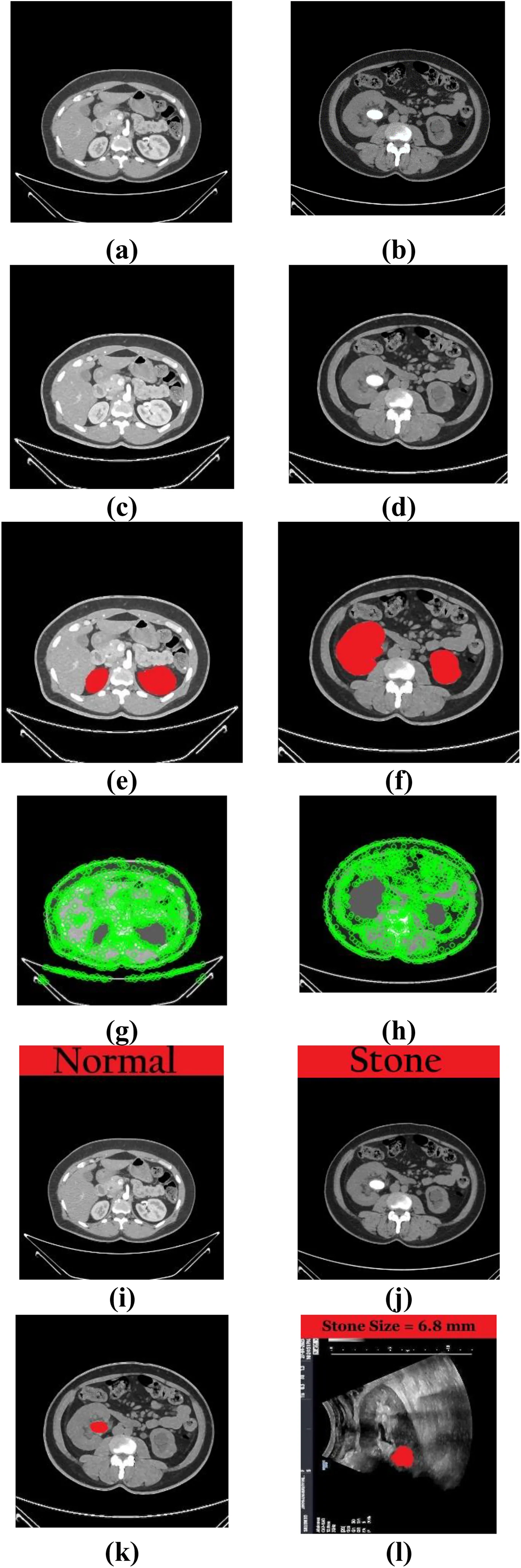
Experimental results of kidney stone detection from KSSD2025 and CT Kidney Dataset, a) input image-1, b) input image-2, c) denoised image-1, d) denoised image-2, e) kidney region segmented image-1, f) kidney region segmented image-2, g) rBRIEF descriptor applied image-1, h) rBRIEF descriptor applied image-2, i) output image-1, j) output image-2, k) stone segmented image, l) kidney stone with size.

### 4.5. Explainability results using Grad-CAM

Grad-CAM is a visualization technique highlighting input image regions that are most influential to the prediction and is applied to both prevailing methods as well as the proposed model to illustrate variations in the areas emphasized. Fig 8 illustrates Explainability utilizing Grad-CAM. Here, dual datasets are considered, and image results with KNN [12], CNN/ResNet [14], PSOWeightedAvgNet [16], Inductive transfer-based ensemble DNN [17], and Proposed MFRANet are given.

**Fig 8:**
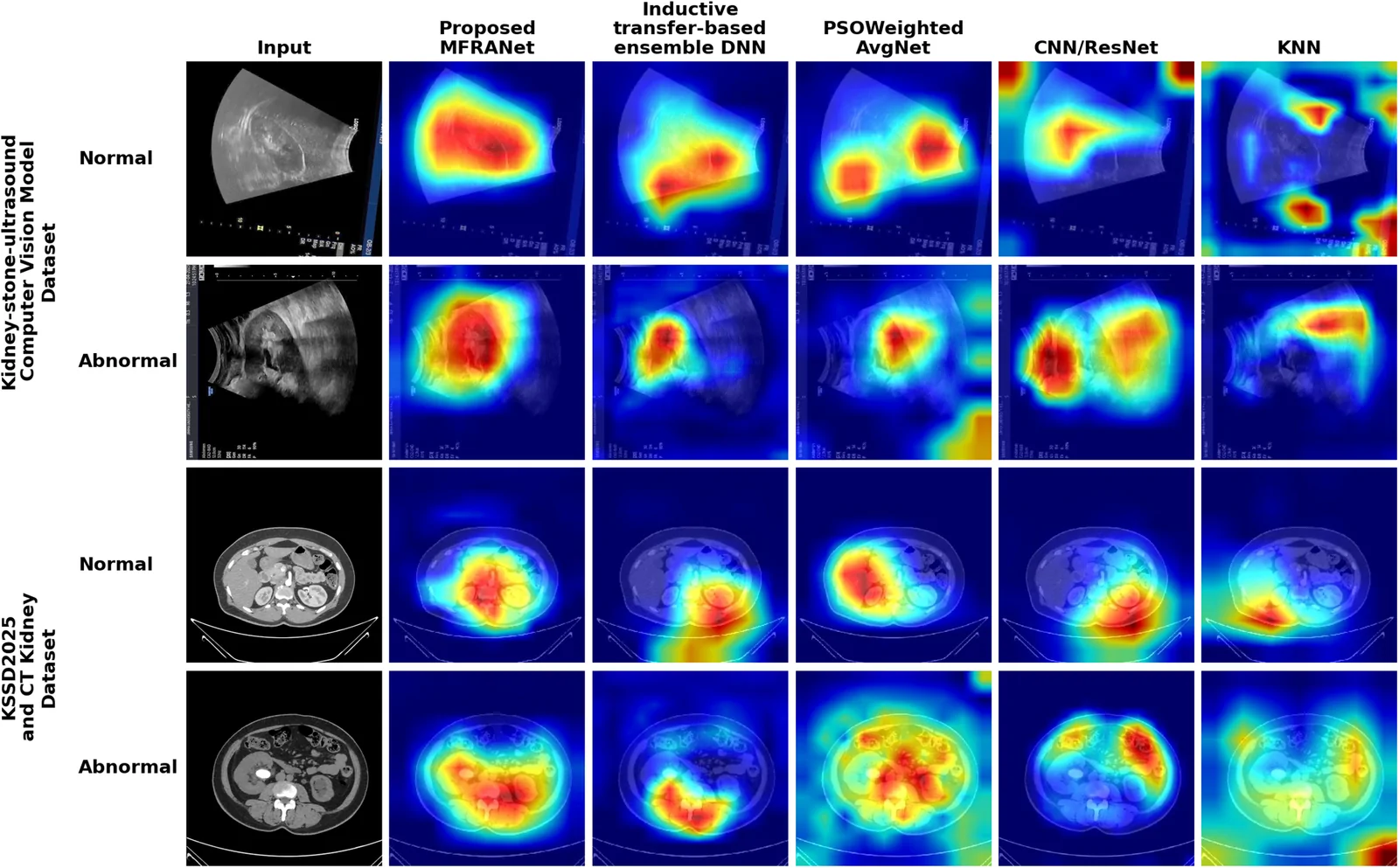
Explainability using Grad-CAM images.

### 4.6. ROC curve

Fig 9 resembles the ROC curve attained with respect to TPR and FPR for KNN [12], CNN/ResNet [14], PSOWeightedAvgNet [16], Inductive transfer-based ensemble DNN [17], and MFRANet. Fig 9a) represents ROC curve based on Kidney-stone-ultrasound Computer Vision Model dataset. For an FPR of 0.8, the TPR is 0.965 for MFRANet, whereas other models gained a TPR of 0.863, 0.880, 0.916, and 0.939. Fig 9b) illustrates ROC curve with respect to KSSD2025 and CT Kidney Dataset. For FPR taken as 0.6, TPR is 0.869, 0.894, 0.918, 0.943, and 0.969 for KNN, CNN/ResNet, PSOWeightedAvgNet, Inductive transfer-based ensemble DNN, and MFRANet. MFRANet’s higher TPR indicates improved kidney stone identification with reduced false detections, due to combined multi-scale attention of MFMANet and contextual learning of GRA-Net.

**Fig 9:**
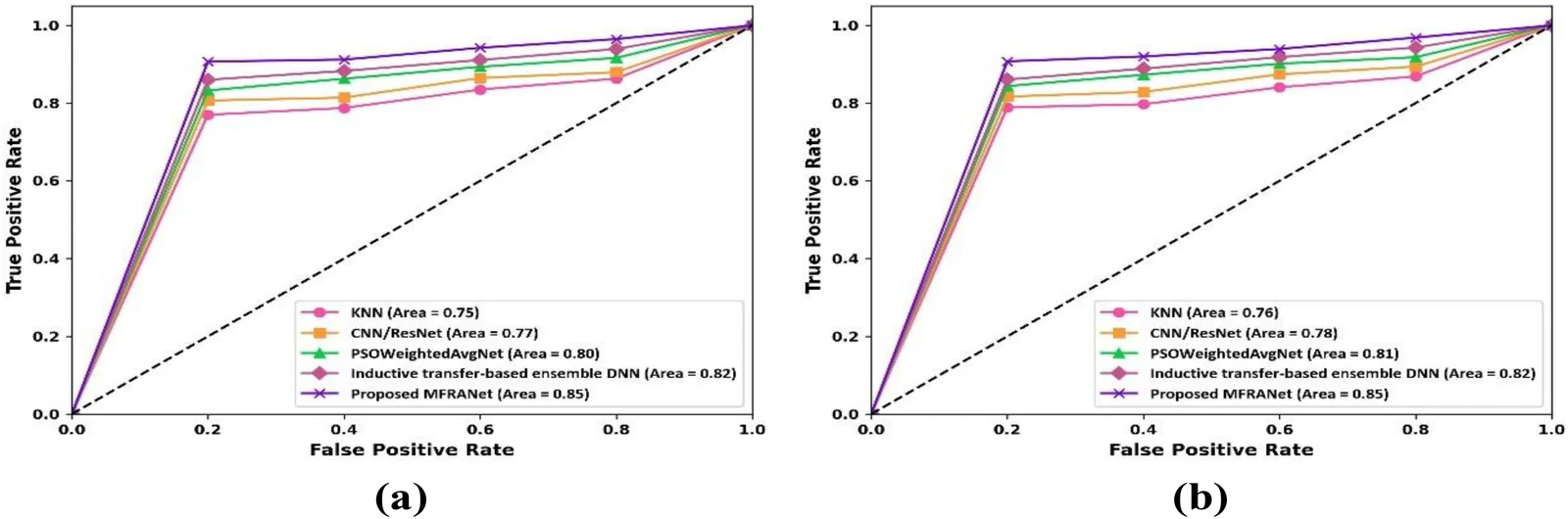
ROC curve, a) Kidney-stone-ultrasound Computer Vision Model dataset, b) KSSD2025 and CT Kidney Dataset.

### 4.7. Ablation study

The ablation study is carried out for two datasets with various methods, like MFMANet [27] +denoising and edge preservation, GRA-Net [28] +denoising and edge preservation, MFRANet, and MFRANet+denoising and edge preservation.

#### 4.7.1. Ablation study based on Kidney-stone-ultrasound Computer Vision Model dataset.

Fig 10 presents an ablation study on the Kidney-stone-ultrasound Computer Vision Model dataset by altering training data from 50% to 90%. Fig 10a) illustrates ablation assessment in terms of accuracy. With 90% training data, MFRANet incorporating MFRANet with denoising and edge preservation achieves the highest accuracy of 96.65%, while baseline methods attain 91.57%, 92.28%, 93.32%, 94.08%, 95.14%, and 95.85%. Fig 10b) presents ablation analysis using F1-score. At training data = 90%, MFRANet achieves highest F1-score of 96.65%, whereas baseline techniques achieve 91.01%, 92.01%, 92.87%, 94.07%, 95.02%, and 95.77%. Fig 10c) depicts an ablation study utilising precision. With 90% training data, MFRANet attains highest precision of 97.44%, while traditional methods record 92.03%, 93.00%, 93.70%, 94.90%, 95.78%, and 96.57%. Fig 10d) presents ablation analysis based on TPR. At 90% training data, MFRANet achieves highest TPR of 95.88%, whereas comparative techniques attain 90.01%, 91.05%, 92.05%, 93.25%, 94.28%, and 94.98%. Overall, the ablation results demonstrate that progressive integration of denoising, edge preservation, and MFRANet consistently enhances accuracy, F1-score, precision, and TPR, thereby confirming the effectiveness of kidney stone detection.

**Fig 10:**
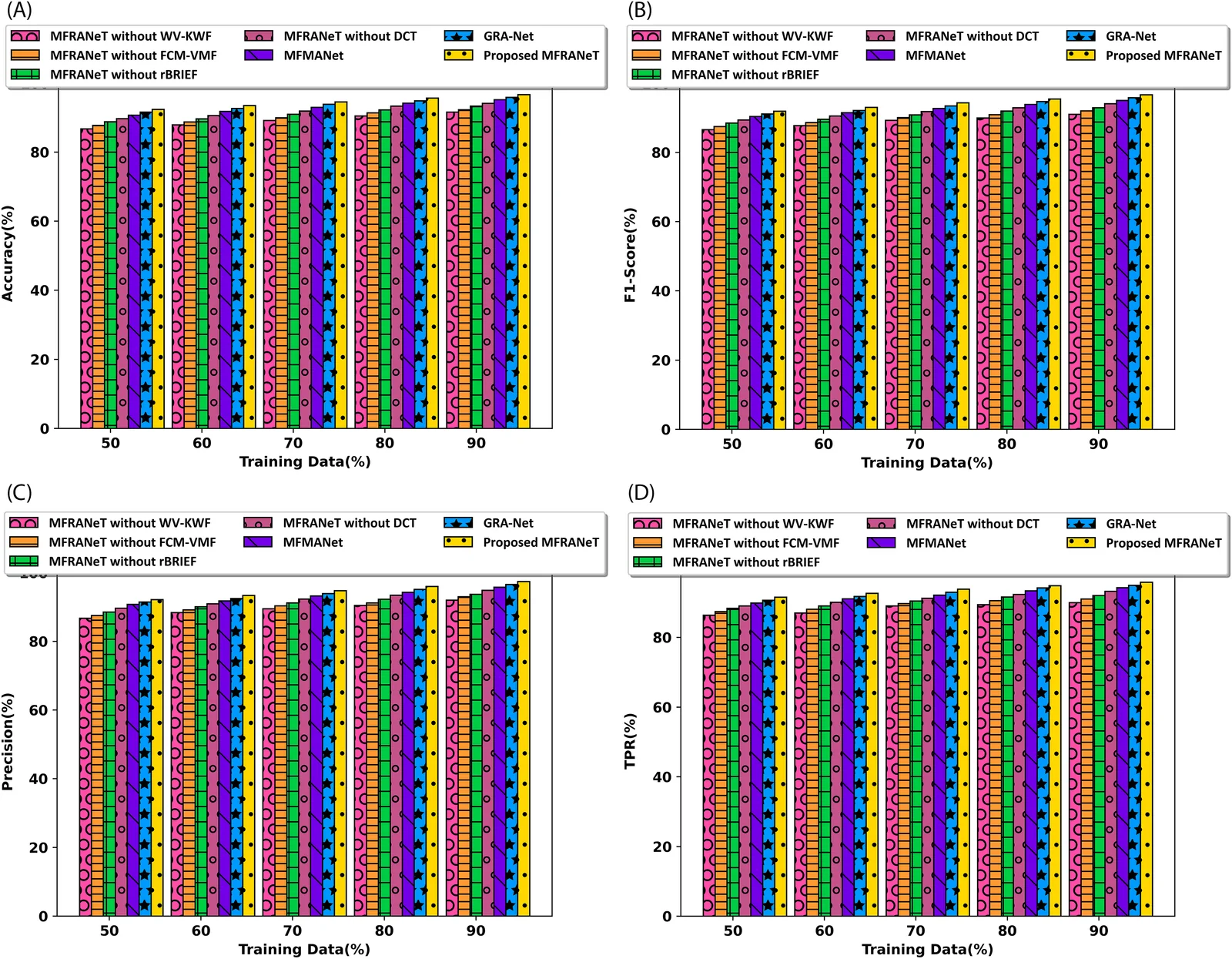
Ablation study with Kidney-stone-ultrasound Computer Vision Model dataset, a) Accuracy, b) F1-score, c) Precision, d) TPR.

#### 4.7.2. Ablation study based on KSSD2025 and CT Kidney Dataset.

Fig 11 presents ablation study conducted on KSSD2025 and CT Kidney datasets to evaluate the effectiveness of MFRANet. Fig 11a) illustrates ablation analysis using accuracy. With training data = 90%, MFRANet with denoising and edge preservation achieves highest accuracy of 96.30%, while baseline methods attain 90.89%, 91.69%, 92.88%, 93.78%, 94.85%, and 95.50%. Fig 11b) presents ablation study based on F1-score. For 90% training data, MFRANet records highest F1-score of 96.39%, whereas comparative methods achieve 91.45%, 92.27%, 93.07%, 94.03%, 94.78%, and 95.52%. Fig 11c) shows ablation analysis in terms of precision. At 90% training data, MFRANet attains highest precision of 97.22%, while baseline methods achieve 91.92%, 92.64%, 93.53%, 94.72%, 95.51%, and 96.34%. Fig 11d) illustrates ablation study using TPR. With 90% training data, MFRANet with denoising and edge preservation achieves highest TPR of 95.59%, whereas traditional methods baseline methods attain 90.99%, 91.90%, 92.61%, 93.35%, 94.07%, and 94.71%. Overall, ablation results demonstrate that integration of denoising, edge preservation, and MFRANet progressively improves feature representation and enhances accuracy, F1-score, precision, and TPR, confirming the effectiveness of MFRANet across KSSD2025 and CT Kidney datasets.

**Fig 11:**
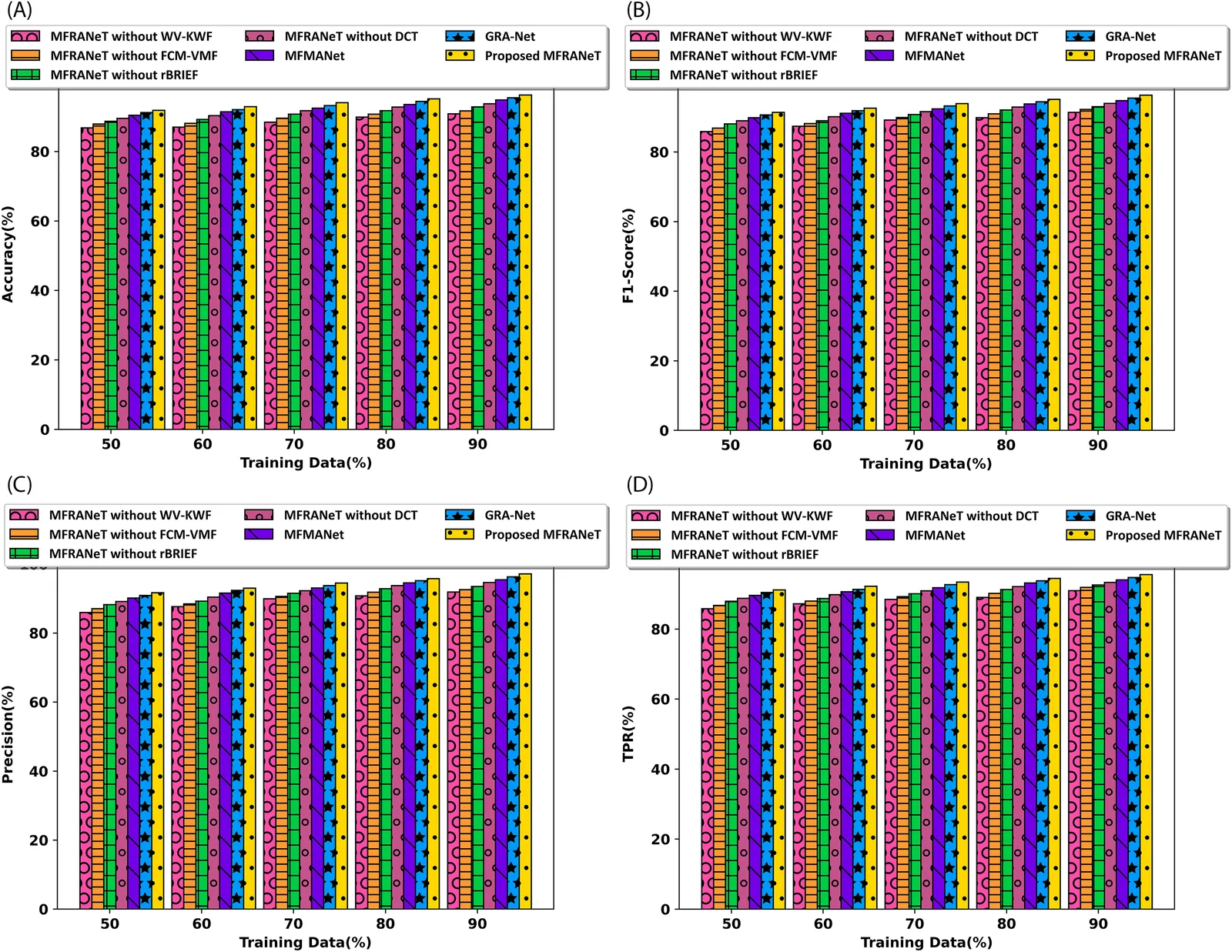
Ablation study with KSSD2025 and CT Kidney Dataset, a) Accuracy, b) F1-score, c) Precision, d) TPR.

### 4.8. Confusion matrix

Fig 12 demonstrates confusion matrix of MFRANet for dual dataset. Fig 12a) represents the confusion matrix of KSSD2025 dataset. Out of 5431 cases, 2995 cases were classified as normal and 78 cases as stones; 111 cases classified normal cases as stones, and 2247 cases classified the stone cases as stones. These results demonstrate the high classification capability of MFRANet with low misclassification. Fig 12b) represents confusion matrix of CT Kidney Dataset. Out of 5915 cases, 801 normal cases are predicted as normal, and 167 stone cases are predicted as normal. 37 normal cases are predicted as stone, and 4910 stone cases are predicted as stone. These results justify the effectiveness of MFRANet, as most normal and stone cases are correctly classified with relatively few misclassifications across both datasets.

**Fig 12:**
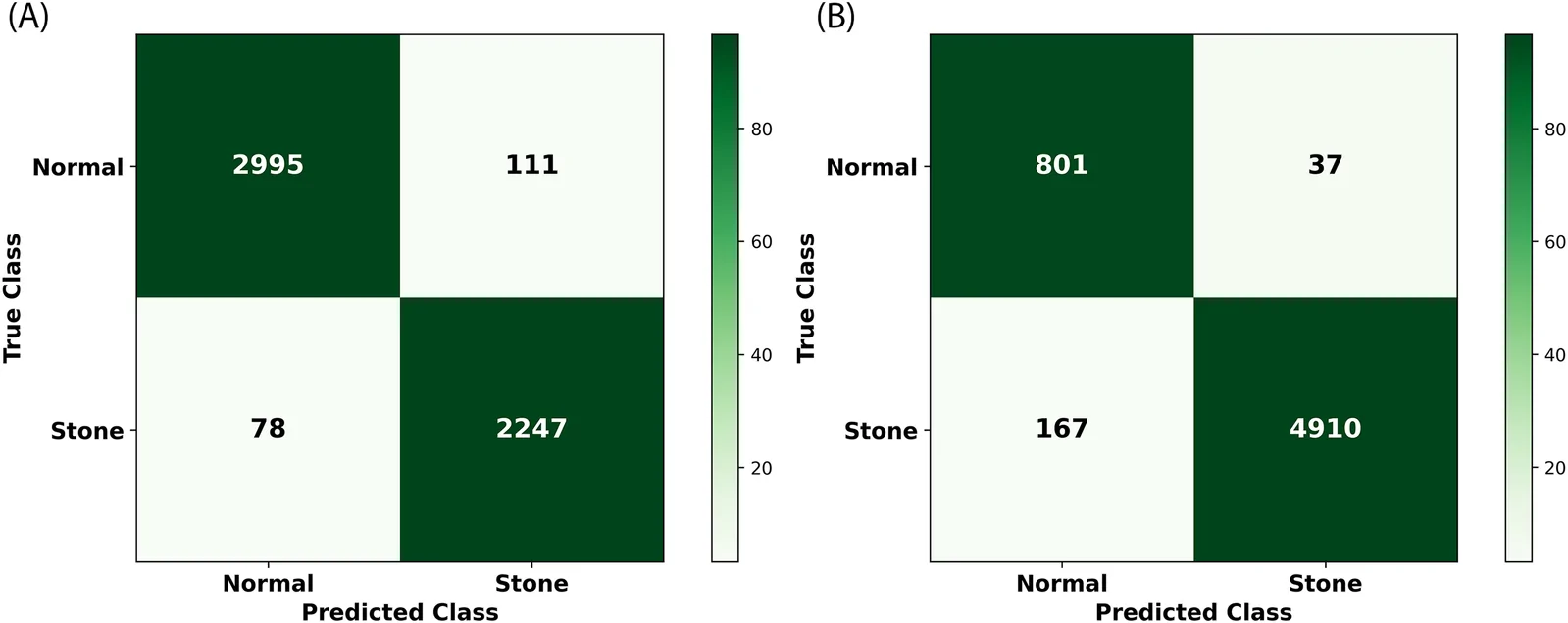
Confusion matrix, a) KSSD2025 dataset, b) CT Kidney Dataset.

### 4.9. Error-case analysis

Fig 13 presents error-case analysis of five methods across six challenging conditions. For small stones, KNN, CNN/ResNet, PSOWeightedAvgNet, Inductive transfer-based ensemble DNN, and MFRANet achieve accuracy of 88.92%, 90.18%, 91.56%, 92.18%, and 94.38%, respectively. For low-contrast images, accuracy obtained by these methods is 84.61%, 86.94%, 88.28%, 90.57%, and 92.12%. Under acoustic shadowing, the methods obtain 86.73%, 88.06%, 90.42%, 91.71%, and 93.18%, respectively. For hydronephrosis, the accuracy is 88.54%, 89.88%, 91.16%, 92.46%, and 94.03%. For cysts/tumors, the respective accuracy is 87.27%, 88.62%, 90.94%, 91.23%, and 93.84%. Finally, for different machines and operators, the methods achieve 89.08%, 90.43%, 91.78%, 93.06%, and 95.56%.

**Fig 13:**
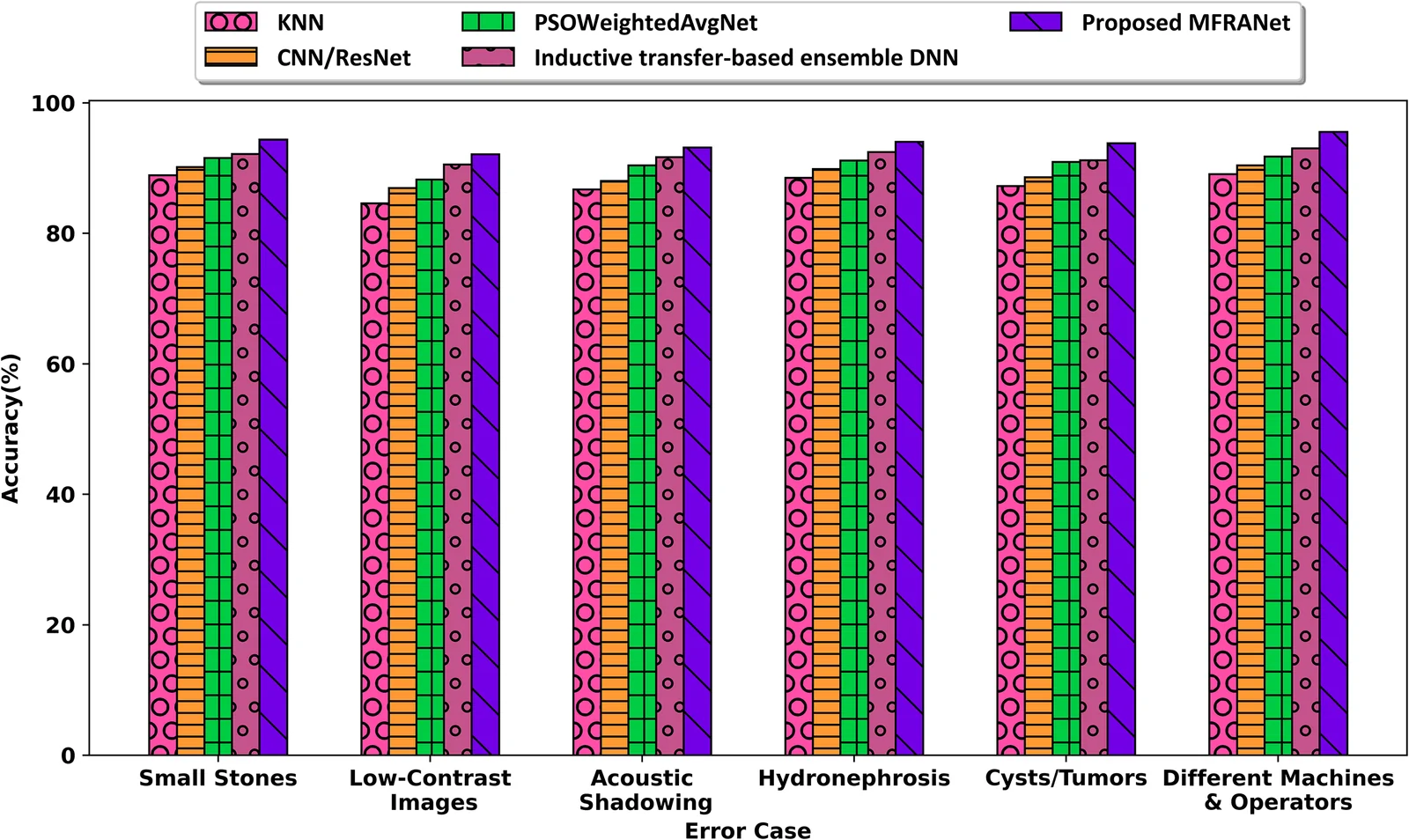
Error-case analysis.

### 4.10. Assessment for Proposed MFRANet with dual datasets

MFRANet is assessed with scalability analysis and cross-fold analysis for kidney stone detection. The methods used for analysis are KNN [12], CNN/ResNet [14], PSOWeightedAvgNet [16], Inductive transfer-based ensemble DNN [17], and Proposed MFRANet. The performance of MFRANet is assessed separately on ultrasound and CT-based datasets to minimize modality-related bias and ensure a fair assessment under different imaging conditions.

#### 4.10.1. Scalability analysis.

Scalability evaluation is employed for kidney stone detection with accuracy, F1-score, precision and TPR based on dual datasets.

a) **Scalability analysis with Kidney-stone-ultrasound Computer Vision Model dataset**

Fig 14 presents scalability analysis with KSSD2025 and CT Kidney Dataset by changing the image size from [32×32] to [512×512]. The image size [512×512] is taken for further analysis. Fig 14a) illustrates scalability assessment based on accuracy. MFRANet achieves accuracy of 96.66%, whereas the baseline methods attain 90.65%, 91.89%, 93.32%, and 94.65% of accuracy. The performance development of MFRANet over other models is 6.01%, 4.77%, 3.34%, and 2.01%. Fig 14b) presents scalability analysis using F1-score. MFRANet achieves highest F1-score of 96.67%, while the other approaches obtain 90.36%, 91.79%, 93.19%, and 94.68%. The performance improvements are 6.31%, 4.88%, 3.48%, and 1.99%. Fig 14c) illustrates scalability assessment based on precision. MFRANet attains highest precision of 97.52%, whereas traditional methods achieve 90.77%, 92.42%, 93.85%, and 95.55%, with improvements of 6.75%, 5.10%, 3.67%, and 1.97%. Fig 14d) shows scalability analysis based on TPR. MFRANet achieves a TPR of 95.83%, while the other methods attain 89.96%, 91.17%, 92.53%, and 93.82%. The corresponding improvements are 5.87%, 4.66%, 3.30%, and 2.01%. Overall, MFRANet maintains superior performance across all evaluation metrics, demonstrating its ability to effectively preserve important spatial and contextual features across different image sizes.

**Fig 14:**
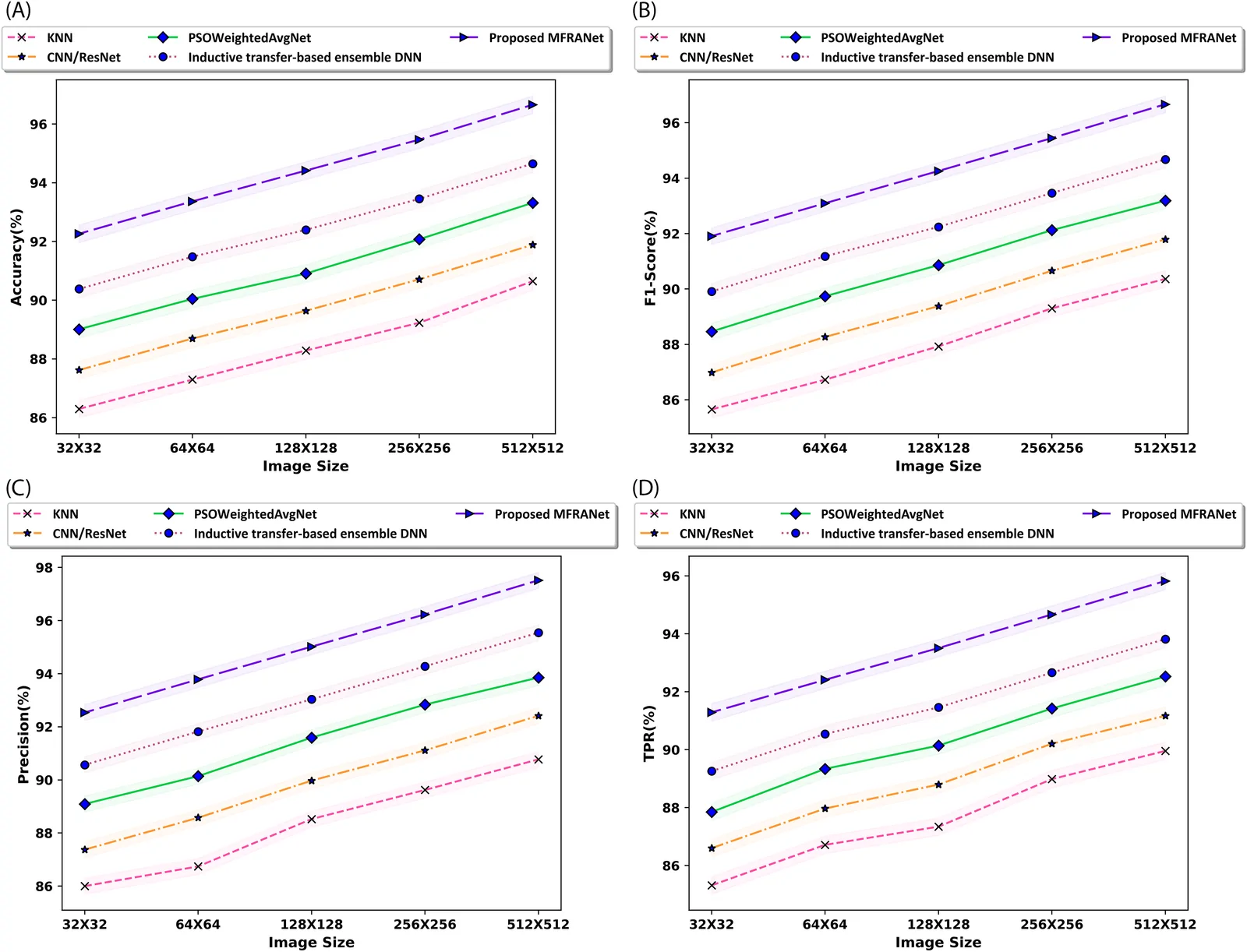
Scalability analysis with Kidney-stone-ultrasound Computer Vision Model dataset, a) Accuracy, b) F1-score, c) Precision, d) TPR.

b) **Scalability analysis with KSSD2025 and CT Kidney Dataset**

Fig 15 presents scalability analysis with the Kidney-stone-ultrasound Computer Vision Model for image size [32×32] to [512×512]. Fig 15a) shows scalability assessment based on accuracy. For an image size of [512×512], accuracy is 96.38% for MFRANet, whereas other approaches achieve 90.02%, 91.43%, 92.93%, and 94.33%, respectively, with performance enhancements of 6.36%, 4.95%, 3.45%, and 2.05%. Fig 15b) specifies scalability assessment using F1-score. MFRANet attains highest F1-score of 96.38%, while other baseline methods achieve 89.66%, 91.16%, 92.80%, and 94.39%, with improvements of 6.72%, 5.22%, 3.58%, and 1.99%. Fig 15c) exemplifies scalability assessment based on precision. MFRANet achieves precision of 97.25%, whereas other approaches attain 90.02%, 91.69%, 93.56%, and 95.29%, with performance improvements of 7.23%, 5.56%, 3.69%, and 1.96%, respectively. Fig 15d) presents scalability analysis based on TPR. MFRANet achieves TPR of 95.53%, while the other models attain 89.31%, 90.64%, 92.05%, and 93.50%, with performance enhancements of 6.22%, 4.89%, 3.48%, and 2.03%, respectively. MFRANet efficiently handles resolution variations by preserving important spatial and contextual information.

**Fig 15:**
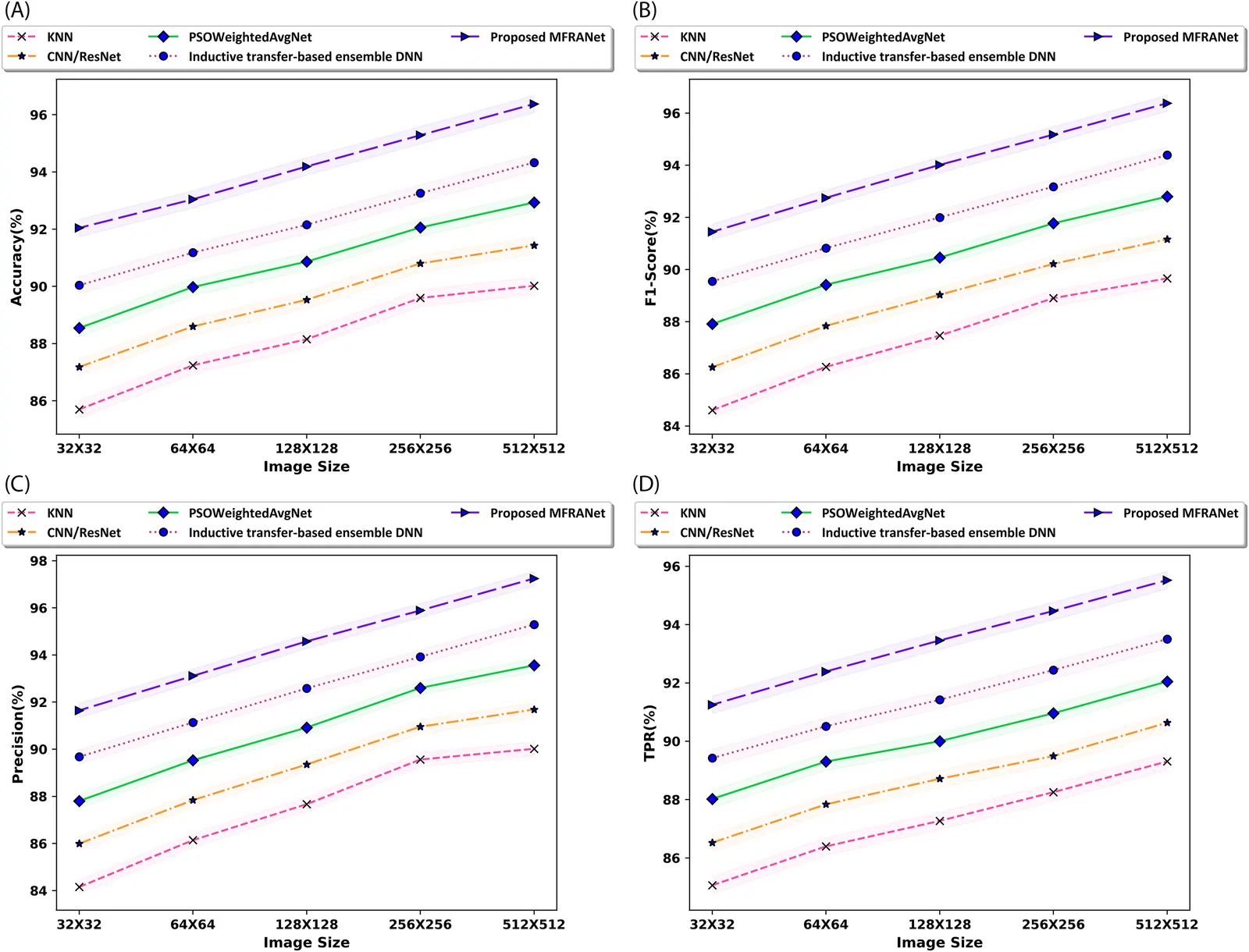
Scalability assessment with KSSD2025 and CT Kidney Dataset, a) Accuracy, b) F1-score, c) Precision, d) TPR.

#### 4.10.2. Cross-fold analysis.

Cross-fold analysis is carried out for kidney stone detection with various metrics based on two datasets.

a) **Cross-fold analysis with Kidney-stone-ultrasound Computer Vision Model dataset**

Fig 16 presents cross-fold analysis with KSSD2025 and CT Kidney Dataset. Fig 16a) shows cross-fold assessment based on accuracy. For a K-fold of 9, accuracy is 96.64% for MFRANet, whereas other approaches attain 90.86%, 92.08%, 93.41%, and 94.63%, respectively. Performance developments of MFRANet over traditional models are 6.36%, 4.56%, 3.23%, and 2.01%. Fig 16b) specifies cross-fold assessment in terms of F1-score. MFRANet achieves highest F1-score of 96.65%, while other approaches obtain 90.11%, 91.65%, 93.10%, and 94.64%, with performance improvements of 6.54%, 5.00%, 3.55%, and 2.01%, respectively. Fig 16c) presents cross-fold assessment based on precision. MFRANet attains a precision of 97.44%, whereas other competitive methods achieve 90.42%, 92.29%, 93.74%, and 95.44%, with improvements of 7.02%, 5.15%, 3.70%, and 2.00%. Fig 16d) illustrates cross-fold assessment based on TPR. MFRANet achieves a TPR of 95.88%, while other comparative approaches attain 89.81%, 91.01%, 92.46%, and 93.86%, with performance enhancements of 6.07%, 4.87%, 3.42%, and 2.02%. Consistent performance across folds demonstrates the generalization capability and reliability of MFRANet for kidney stone detection.

**Fig 16:**
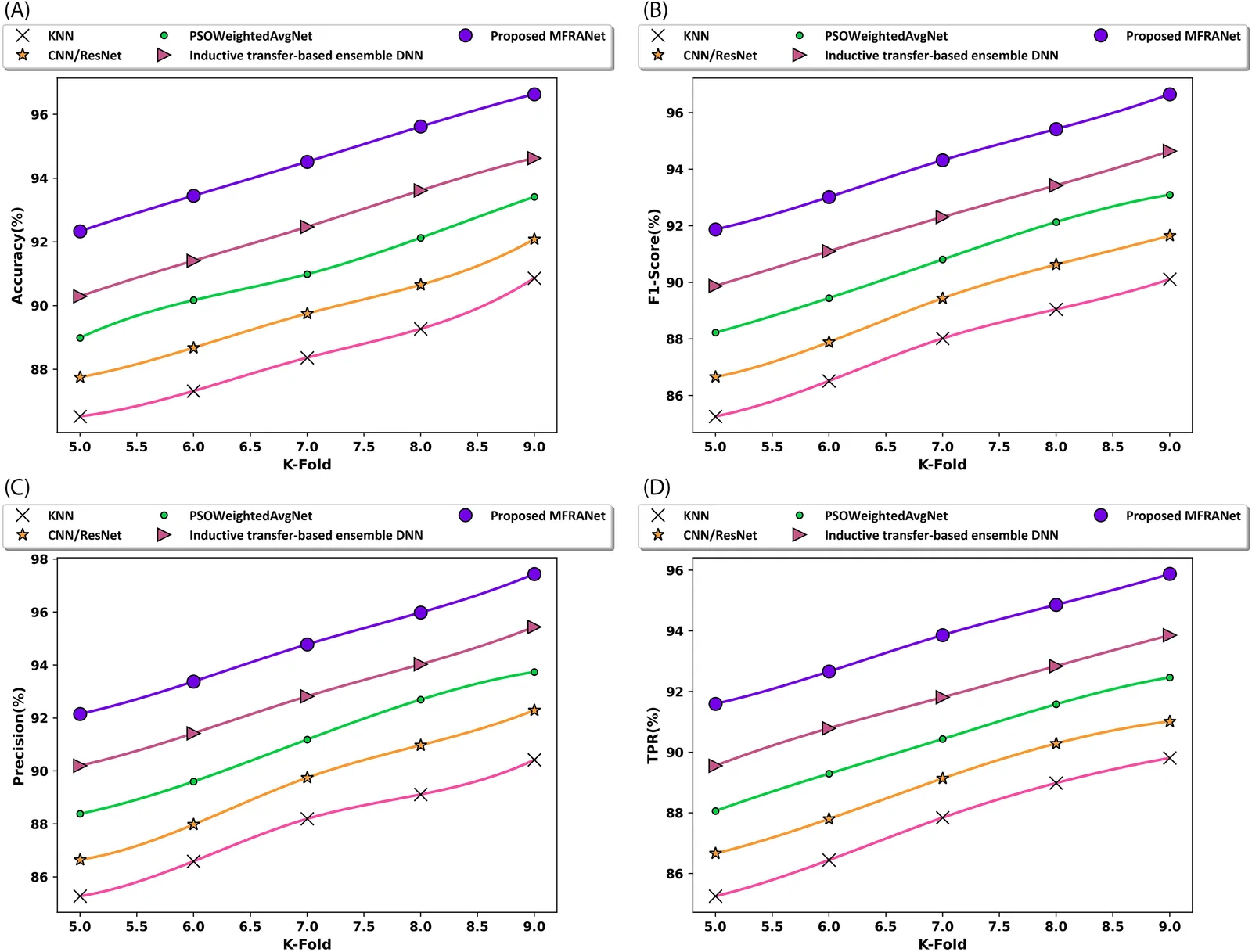
Cross-fold analysis with Kidney-stone-ultrasound Computer Vision Model dataset, a) Accuracy, b) F1-score, c) Precision, d) TPR.

b) **Cross-fold analysis with KSSD2025 and CT Kidney Dataset**

Fig 17 illustrates cross-fold analysis with Kidney-stone-ultrasound Computer Vision Model dataset. Fig 17a) shows cross-fold assessment on accuracy. For a K-fold of 9, accuracy is 96.30% for MFRANet, whereas comparative methods attain 90.28%, 91.52%, 92.88%, and 94.30%, respectively, with performance enhancements of 6.02%, 4.78%, 3.42%, and 2.00%. Fig 17b) specifies cross-fold assessment in terms of F1-score. MFRANet achieves highest F1-score of 96.38%, while baseline techniques obtain 89.91%, 91.35%, 92.87%, and 94.36%, with performance improvements of 6.47%, 5.03%, 3.51%, and 2.02%. Fig 17c) presents cross-fold assessment using precision. MFRANet attains precision of 97.20%, whereas the traditional methods achieve 90.50%, 92.01%, 93.64%, and 95.20%, with improvements of 6.70%, 5.19%, 3.56%, and 2.00%, respectively. Fig 17d) illustrates cross-fold assessment based on TPR. MFRANet achieves TPR of 97.20%, while competitive approaches attain 90.50%, 92.01%, 93.64%, and 95.20%, with performance improvements of 6.70%, 5.19%, 3.56%, and 2.00%. The consistent performance across folds demonstrates the generalization capability and reliability of MFRANet for kidney stone detection.

**Fig 17:**
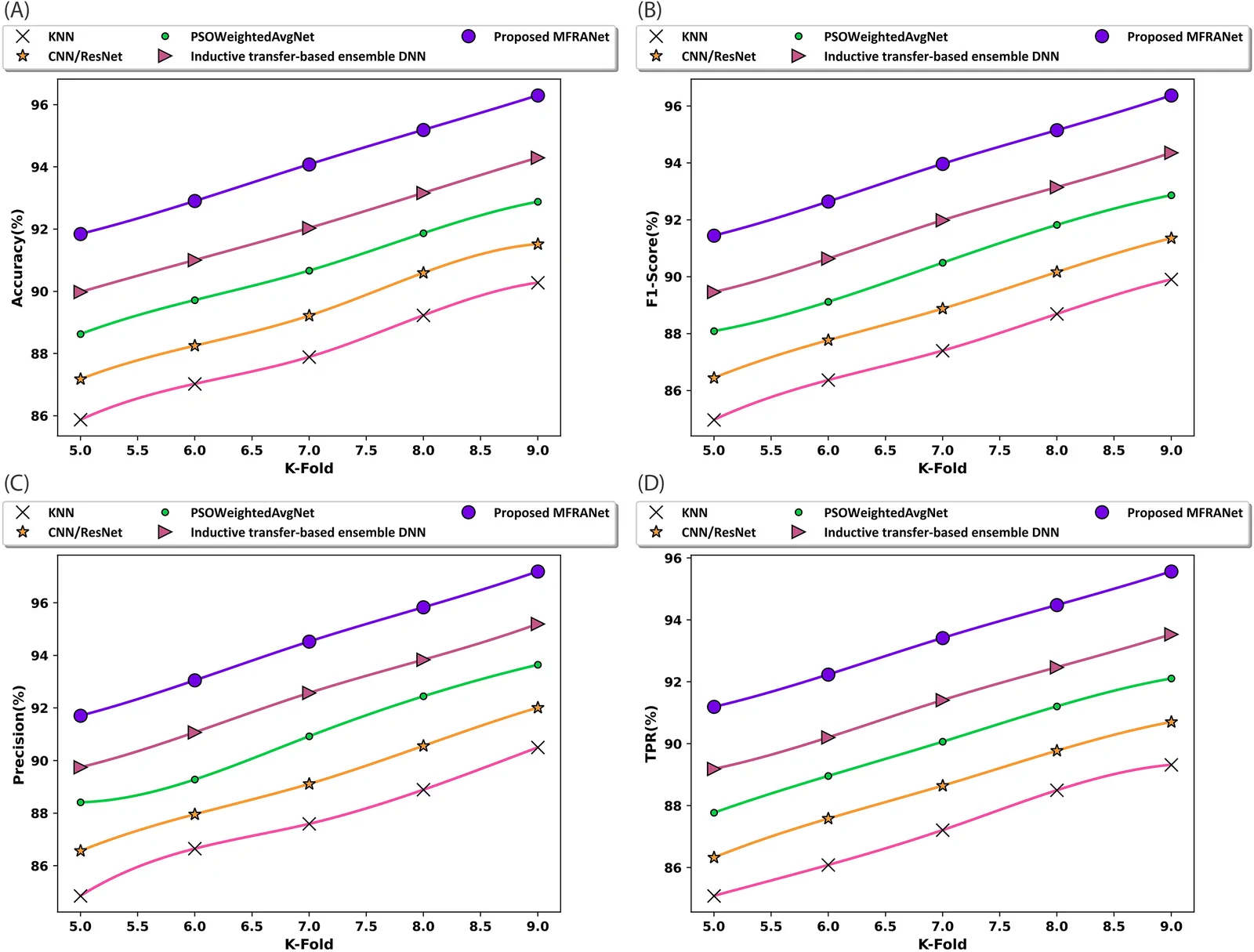
Cross-fold analysis with KSSD2025 and CT Kidney Dataset, a) Accuracy, b) F1-score, c) Precision, d) TPR.

### 4.11. Discussion

Results achieved from scalability analysis, cross-fold assessment, and Wilcoxon test are discussed below. The obtained results are analyzed separately for ultrasound and CT-based datasets to verify the robustness of MFRANet across different imaging modalities.

#### 4.11.1. Wilcoxon test.

Table 6 implies the Wilcoxon test-based discussion. Wilcoxon test value less than 0.05 is indicated as statistically significant. To find the statistical significance of MFRANet performance over other models, Wilcoxon test is employed on accuracy and TPR across 5-fold cross-validation with two datasets. In Kidney-stone-ultrasound Computer Vision Model dataset, every comparison yielded statistically significant outputs (p < 0.05), with MFRANet showing superior accuracy than KNN (W = 2.611, p = 0.0294), CNN/ResNet (W = 2.106, p = 0.0265), PSOWeightedAvgNet (W = 1.355, p = 0.0138), and Inductive transfer-based ensemble DNN (W = 1.009, p = 0.0065). Results are further confirmed on KSSD2025 and CT Kidney Dataset with respect to accuracy and TPR.

**Table 6:** Wilcoxon test-based discussion.

Proposed	Comparative methods	Kidney-stone-ultrasound Computer Vision Model dataset				KSSD2025 and CT Kidney Dataset			
		W-statistic		P-Value		W-statistic		P-Value	
**MFRANet**		**ACC**	**TPR**	**ACC**	**TPR**	**ACC**	**TPR**	**ACC**	**TPR**
	**KNN** [12]	2.611	2.117	0.0294	0.0307	2.579	2.189	0.0299	0.0298
	**CNN/ResNet** [14]	2.106	1.658	0.0265	0.0240	2.143	1.642	0.0297	0.0246
	**PSOWeightedAvgNet** [16]	1.355	1.515	0.0138	0.0130	1.124	1.275	0.0139	0.0139
	**Inductive transfer-** **based ensemble DNN** [17]	1.009	1.050	0.0065	0.0117	1.141	1.085	0.0076	0.0113

#### 4.11.2. Comparative discussion.

Table 7 signifies comparative discussion of MFRANet with other techniques. When Kidney-stone-ultrasound Computer Vision Model dataset is considered, accuracy attained is 96.64% for MFRANet, whereas other models attained accuracies of 90.86%, 92.08%, 93.41%, and 94.63% for K-fold analysis with a K-fold value of 9. Similarly, MFRANet achieved an F1-score of 96.65%, precision of 97.44%, and TPR of 95.88%, whereas other models attained F1-scores of 90.11%, 91.65%, 93.10%, and 94.64%, precisions of 90.42%, 92.29%, 93.74%, and 95.44%, and TPR values of 89.81%, 91.01%, 92.46%, and 93.86%, respectively. Also, for K-fold analysis done with respect to the Kidney-stone-ultrasound Computer Vision Model dataset, accuracy and TPR achieved are 96.64% and 95.88%, respectively. In case of scalability analysis, for assessment with the Kidney-stone-ultrasound Computer Vision Model dataset, accuracy and TPR achieved are 96.38% and 95.53% when image size is [512×512]. Also, for assessment with respect to KSSD2025 and CT Kidney Dataset, accuracy and TPR are 96.66% and 95.83% when image size is [512×512]. Hence, maximal metric values are attained when image is assessed using maximal K-fold value. Hence, the newly formed combined network MFRANet enhanced the ability of focusing on relevant spatial and contextual features, thereby improving discriminative capability and reducing false detections. Also, MFRANet is capable of handling real-world kidney stone detection tasks with high reliability and consistency.

**Table 7:** Comparative discussion.

Analysis	Datasets	Methods/Metrics	KNN	CNN/ResNet	PSO Weighted AvgNet	Inductive transfer-based ensemble DNN	Proposed MFRANet
**Scalability analysis** **(Image size=** [512×512])	**Kidney-stone-ultrasound Computer Vision Model dataset**	***Accuracy (%)***	90.65	91.89	93.32	94.65	96.66
		***F1-score (%)***	90.36	91.79	93.19	94.68	96.67
		***Precision (%)***	90.77	92.42	93.85	95.55	97.52
		***TPR (%)***	89.96	91.17	92.53	93.82	95.83
	**KSSD2025 and CT Kidney Dataset**	***Accuracy (%)***	90.02	91.43	92.93	94.33	96.38
		***F1-score***	89.66	91.16	92.80	94.39	96.38
		***Precision***	90.02	91.69	93.56	95.29	97.25
		***TPR (%)***	89.31	90.64	92.05	93.50	95.53
**K-fold analysis (K-fold = 9)**	**Kidney-stone-ultrasound Computer Vision Model dataset**	***Accuracy (%)***	90.86	92.08	93.41	94.63	**96.64**
		***F1-score (%)***	90.11	91.65	93.10	94.64	**96.65**
		***Precision (%)***	90.42	92.29	93.74	95.44	**97.44**
		***TPR (%)***	89.81	91.01	92.46	93.86	**95.88**
	**KSSD2025 and CT Kidney Dataset**	***Accuracy (%)***	90.28	91.52	92.88	94.30	96.30
		***F1-score (%)***	89.91	91.35	92.87	94.36	96.38
		***Precision (%)***	90.50	92.01	93.64	95.20	97.20
		***TPR (%)***	90.50	92.01	93.64	95.20	97.20

### 4.12. Confidence Intervals using Kidney-stone-ultrasound Computer Vision Model dataset and KSSD2025 and CT Kidney Dataset

Table 8 presents the confidence intervals (CIs) for accuracy, F1-score, precision, and TPR of the evaluated methods. MFRANet achieves the highest performance ranges across all four metrics, with accuracy of 92.08–96.88%, F1-score of 91.54–96.90%, precision of 91.87–97.68%, and TPR of 91.32–96.08%. In comparison, KNN, CNN/ResNet, PSOWeightedAvgNet, and Inductive transfer-based ensemble DNN show progressively lower confidence intervals. The higher and consistently strong CIs of MFRANet indicate its superior and stable classification performance compared with the competing methods.

**Table 8:** Confidence Intervals based on Kidney-stone-ultrasound Computer Vision Model dataset.

Method	Accuracy	F1-score	Precision	TPR
KNN	86.16–91.11	84.99–90.32	84.91–90.64	84.96–90.05
CNN/ResNet	87.48–92.36	86.29–91.90	86.31–92.58	86.46–91.29
PSOWeightedAvgNet	88.74–93.69	87.93–93.34	88.04–93.96	87.81–92.71
Inductive transfer-based ensemble DNN	90.01–94.85	89.65–94.84	89.91–95.73	89.19–94.10
Proposed MFRANet	92.08–96.88	91.54–96.90	91.87–97.68	91.32–96.08

Table 9 presents the confidence intervals for accuracy, F1-score, precision, and TPR on KSSD2025 and CT Kidney Dataset. MFRANet achieves the highest confidence intervals across all metrics, with accuracy of 91.47–96.51%, F1-score of 91.21–96.59%, precision of 91.45–97.49%, and TPR of 90.80–95.83%. The competing methods show comparatively lower ranges, with Inductive transfer-based ensemble DNN providing the next-best results. These results demonstrate the consistent and superior performance of MFRANet on KSSD2025 and CT Kidney Dataset.

**Table 9:** Confidence Intervals based on KSSD2025 and CT Kidney Dataset.

Method	Accuracy	F1-score	Precision	TPR
KNN	85.50–90.58	84.75–90.11	84.51–90.70	84.74–89.58
CNN/ResNet	86.94–91.80	86.15–91.62	86.33–92.26	85.93–90.97
PSOWeightedAvgNet	88.26–93.08	87.80–93.10	88.19–93.85	87.49–92.37
Inductive transfer-based ensemble DNN	89.62–94.54	89.17–94.62	89.52–95.46	88.90–93.75
Proposed MFRANet	91.47–96.51	91.21–96.59	91.45–97.49	90.80–95.83

## 5. Conclusion

Kidney stone formation occurs when dissolved minerals in urine crystallize and aggregate within the renal calyces or pelvis. The clinical importance of early stone detection lies in preventing urinary tract obstruction, infection, and renal impairment. Ultrasound-based detection is widely adopted for its safety and accessibility, yet its diagnostic precision is limited by speckle noise, poor contrast, and operator dependency. Therefore, developing advanced computational models for automatic stone detection and segmentation is vital to enhance diagnostic accuracy. To address these limitations, MFRANet is proposed for efficient detection of kidney stone. Initially, input ultrasound images undergo denoising and edge preservation using WV-KWF. Kidney region is then segmented with FCM-VMF. Subsequently, feature descriptors are extracted using the rBRIEF method combined with DCT. Kidney stone detection is then performed using MFRANet merges the strengths of MFMANet and GRA-Net. Lastly, detected stones are segmented by O-DAT, and the stone size is measured. MFRANet shows superior performance, attaining accuracy of 96.64% and TPR of 95.88%. Future research can focus on integrating large and more diverse ultrasound datasets to improve generalization of the model across different imaging devices.
